# Field Studies Reveal Strong Postmating Isolation between Ecologically Divergent Butterfly Populations

**DOI:** 10.1371/journal.pbio.1000529

**Published:** 2010-10-26

**Authors:** Carolyn S. McBride, Michael C. Singer

**Affiliations:** 1 Center for Population Biology, University of California Davis, Davis, California, United States of America; 2 Section of Integrative Biology, University of Texas, Austin, Texas, United States of America; Duke University, United States of America

## Abstract

Gene flow between populations that are adapting to distinct environments may be restricted if hybrids inherit maladaptive, intermediate phenotypes. This phenomenon, called extrinsic postzygotic isolation (EPI), is thought to play a critical role in the early stages of speciation. However, despite its intuitive appeal, we know surprisingly little about the strength and prevalence of EPI in nature, and even less about the specific phenotypes that tend to cause problems for hybrids. In this study, we searched for EPI among allopatric populations of the butterfly *Euphydryas editha* that have specialized on alternative host plants. These populations recall a situation thought typical of the very early stages of speciation. They lack consistent host-associated genetic differentiation at random nuclear loci and show no signs of reproductive incompatibility in the laboratory. However, they do differ consistently in diverse host-related traits. For each of these traits, we first asked whether hybrids between populations that use different hosts (different-host hybrids) were intermediate to parental populations and to hybrids between populations that use the same host (same-host hybrids). We then conducted field experiments to estimate the effects of intermediacy on fitness in nature. Our results revealed strong EPI under field conditions. Different-host hybrids exhibited an array of intermediate traits that were significantly maladaptive, including four behaviors. Intermediate foraging height slowed the growth of larvae, while intermediate oviposition preference, oviposition site height, and clutch size severely reduced the growth and survival of the offspring of adult females. We used our empirical data to construct a fitness surface on which different-host hybrids can be seen to fall in an adaptive valley between two peaks occupied by same-host hybrids. These findings demonstrate how ecological selection against hybrids can create a strong barrier to gene flow at the early stages of adaptive divergence.

## Abstract 2

A mismatch between hybrid butterflies and their ecological environment restricts gene flow between populations that feed on different host plants, highlighting the potential importance of a seldom-studied mechanism of reproductive isolation.

## Author Summary

When two populations adapt to different ecological environments, they may become reproductively incompatible with each other and eventually form distinct species. One form of incompatibility thought to contribute to this process occurs when hybrids between diverging populations are ecologically maladapted. They suffer reduced survival and reproduction because they possess intermediate traits that are ill-suited to both parental environments. Although this phenomenon is potentially important at the early stages of speciation, it is difficult to study in the field and is often invisible in the laboratory—leaving us with few empirical examples. We use a series of behavioral assays and manipulative field experiments to examine hybrids between populations of a butterfly that have adapted to use distinct host plants. We show that the hybrids are perfectly healthy in the laboratory. However, when taken into the field, they interact with their host plants in intermediate and anomalous ways that lower the growth and survival of both themselves and their offspring. Our findings confirm that ecological selection against hybrids has great potential to block gene flow at the early stages of adaptive divergence.

## Introduction

The idea that ecological divergence can drive speciation has been discussed, studied, and widely accepted since the time of Darwin [1]–. It is thus surprising that we know so little about one of the most important mechanisms by which ecological divergence may contribute to species formation. Imagine two populations adapting to different ecological niches. Such adaptation will reduce gene flow between the populations if hybrids have intermediate phenotypes that fare poorly in both parental habitats—in essence, if hybrids fall in an adaptive valley between the fitness peaks associated with the niches of their parents. This phenomenon is called extrinsic postzygotic isolation (EPI) because it obstructs gene flow after hybrid zygotes have formed (hence postzygotic) and arises from an interaction between hybrids and their environment rather than from inherent developmental defects (hence extrinsic).

Verbal theory and intuition suggest that EPI may be a widespread and important component of speciation. For one, it is expected to develop early, at the onset of phenotypic divergence. Barriers that develop early are critical to speciation because they can facilitate subsequent divergence and speed the accumulation of additional forms of isolation. Early barriers that are postzygotic, in particular, can lead to direct selection for assortative mating via reinforcement [2],[10]. Second, EPI has the potential to act in any system involving ecological divergence, regardless of its biological particulars. This sets it apart from several forms of prezygotic isolation, which can only contribute to speciation when the alternative niches are directly linked to mate choice (e.g., habitat isolation, temporal isolation, pollinator isolation, and sexual isolation based on ecological traits that double as mating cues [6],[8]).

Given the potential importance and generality of extrinsic postzygotic isolation, many have lamented how little we know about its prevalence, strength, and character in nature (e.g., [6]–[8],[11]). It has received much less empirical attention than any other major form of reproductive isolation, perhaps because its ephemeral nature makes it difficult to study. The clearest examples in which ecological selection against hybrids has been established and quantified include benthic and limnetic morphs of the threespine stickleback [12]–[15], large and small billed *Geospiza* ground finches [16],[17], basin and mountain subspecies of big sagebrush [18], mimetic races of *Heliconius* butterflies [19], and *Neochlamisus* leaf beetle host races [20] (see also [21]–[30]). In host races of *Rhagoletis* fruit flies [31],[32] hybrids possess intermediate traits that are almost certain to be maladaptive, though the effect has not been directly quantified (see also [33]). While these studies confirm the existence of extrinsic postzygotic isolation in natural systems, major gaps in understanding remain.

One gap involves the relative importance of EPI to other ecological forms of isolation known to contribute to speciation. As noted above, prezygotic isolation can provide a potent barrier to gene flow when divergent selection acts directly on characters that influence mate choice [6]–[8]. Such mating barriers are better documented than EPI and appear to be stronger than EPI in the systems where EPI has been shown to exist (e.g., sexual isolation in sticklebacks, Darwin's finches, and *Heliconius* butterflies [19],[34]–[36]; habitat isolation in *Neochlamisus* leaf beetles and other insect host races [20],[23],[37]). We are aware of no cases where ecological selection against hybrids is the primary isolating factor between diverging populations and it remains possible that it is often incidental to speciation. Or perhaps its most important contribution comes at an early stage of divergence that has rarely been examined—among allopatric populations at or before the time they come into contact in sympatry.

We also know relatively little about the mechanistic bases of EPI. The most common empirical approach to studying the phenomenon focuses on quantifying it and distinguishing it from intrinsic postzygotic isolation, where hybrids perform poorly due to inherent developmental defects. Postzygotic isolation is detected by showing that hybrids are less fit than their parents in the specific environments to which the parents are adapted. Confirmation that the isolation is extrinsic is then achieved by showing that the fitness deficit disappears in a “neutral” environment [12] or, more rigorously, by showing that the rank order of fitness of the two backcross types switches between parental environments [14],[38]. This approach has the advantage of assaying fitness directly in hybrid individuals, but it does not reveal which interactions between hybrids and their environment are dysfunctional. Are certain types of traits (e.g., morphological, physiological, or behavioral) more likely to lead to hybrid dysfunction than others?

A third gap in current knowledge of EPI is that, with a few notable exceptions (e.g., [15]–[17]), existing estimates of its strength come from laboratory environments [20],[23],[25],[27],[29],[30] or from modified field environments (e.g., enclosures that exclude predators/grazers [12]–[14],[18],[21]). There are obvious reasons for this practice. Tracking individual organisms and measuring their fitness in nature is difficult, if not impossible, in most systems. Yet fitness estimates that come from artificial environments will only be accurate to the extent that key aspects of the ecological niches in question have been replicated. For example, bringing the alternative host plants of diverging insect populations into the lab or greenhouse allows researchers to evaluate the costs hybrids face due to intermediate digestive physiology but will likely miss those associated with factors such as host-specific predators, pathogens, and microclimates. The scarcity of field studies raises the possibility that we have systematically underestimated the true strength of extrinsic postzygotic isolation in nature.

**Figure 1:**
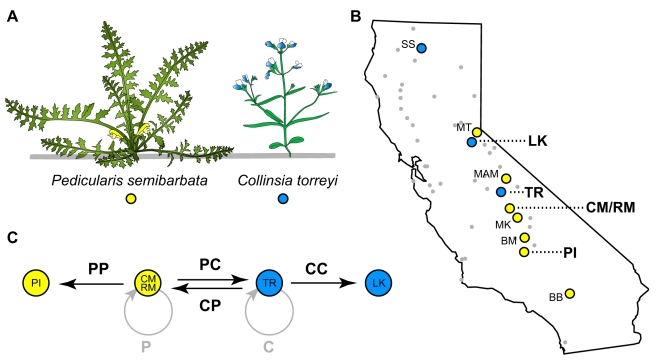
Study system background and experimental design. (A) Sketches of the two host plants addressed in this study: *Pedicularis semibarbata* (*Psem*, Orobanchaceae), a perennial herb with basal rosette morphology, and *Collinsia torreyi* (*Ctor*, Plantaginaceae), a small upright annual herb. (B) Map of California highlighting *E. editha* populations that use *Ctor* (blue circles), *Psem* (yellow circles), or other plants (grey dots) as primary hosts (see [39] for a description of patterns of host use in this species overall). This study specifically addresses the four populations in bold: Leek Spring (LK), Tamarack Ridge (TR), Colony Meadow/Rabbit Meadow metapopulation (CM/RM), and Piute Mountain (PI). (C) Schematic of the crosses performed to produce insects examined in this study. Colored circles represent populations from which parents were sampled. Each black arrow represents a specific type of cross and leads from the native population of the female to the native population of her male mate. Bold black two-letter symbols above or below arrows symbolize the offspring produced by the cross, the first and second letters corresponding to the host affiliations of the mother and father, respectively (P, *Psem*; C, *Ctor*). Thus, the left-most arrow indicates that females from the *Psem*-adapted CM population were mated to males from the *Psem*-adapted PI population to produce PP offspring. Likewise, the middle arrows show the crosses used to produce PC offspring (with a *Psem*-adapted mother and *Ctor*-adapted father) and CP offspring (reciprocal of the previous). Grey circular arrows represent within population crosses that were not conducted by us but occurred naturally prior to the capture of mated females. These crosses produced “pure” offspring symbolized as simply P or C.

**Figure 2:**
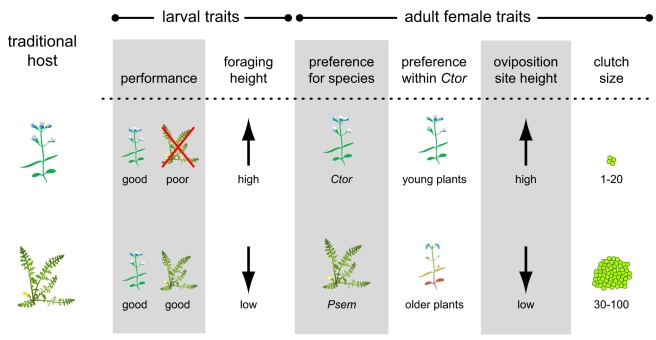
Summary of traits addressed in this study. *E. editha* populations adapted to *Collinsia torreyi* (top row) and *Pedicularis semibarbata* (bottom row) have diverged in the six traits illustrated—two expressed in larvae and four expressed in adult females [40]. Larval performance: Both types of larvae grow and survive well on *Ctor*, but only *Psem*-adapted larvae are able to survive on *Psem*. Larval foraging height: *Ctor*- and *Psem*-adapted larvae tend to feed at the top and base of their hosts, respectively. Female oviposition preference: Adult females prefer to lay eggs on the host to which they are adapted. Moreover, when forced to lay on *Ctor*, *Ctor*- and *Psem*-adapted females prefer individual plants at earlier and later phenological stages, respectively. Oviposition site height: *Ctor*-adapted females tend to lay their eggs at the top of their host near the point where they first land. *Psem*-adapted females invariably drop to explore the basal leaves of their host and lay near the ground. Clutch size: *Ctor*-adapted females lay 1–20 eggs per clutch while *Psem*-adapted females lay 30–100 eggs per clutch. This does not translate into a difference in either daily or lifetime fecundity since *Ctor*-adapted females lay more frequently than *Psem*-adapted females. No major phenotypic differences have been described in pupae or adult males.

The checkerspot butterfly *Euphydryas editha* provides a tractable system in which to begin addressing these gaps in understanding. The species is made up of multiple, allopatric populations in various stages of adaptation to distinct host plants [39]. Those populations adapted to *Collinsia torreyi* and *Pedicularis semibarbata* (Figure 1A) are distributed along the western slope of the Sierra Nevada in California (Figure 1B) in patches of coniferous woodland habitat where the two host plants are intermingled at the scale of inches to feet [40]. Despite host sympatry, the butterflies at any given site have evolved to lay eggs on just one of the two plant species and ignore the other, with the identity of the used host flip-flopping back and forth between sites (Figure 1B). The populations that use different hosts have diverged in several important host-related traits, ranging from larval performance and foraging height to adult female host preference, oviposition site height, and clutch size (Figure 2) [40]. Consistent host-associated differences in morphology and random genetic markers, on the other hand, remain elusive. A previous examination of >400 AFLPs revealed significant genetic differentiation among populations [39],[41], but this differentiation was not associated with the use of the two host plants examined here (subset of analyses from [41] summarized in Text S1).

Here, we obtain field-based estimates of the strength of extrinsic postzygotic isolation in this system and describe its underlying mechanisms. For one trait known to have diverged among populations, we directly measure the fitness of hybrids expressing that trait in the field. For the five remaining traits, we first determine the extent to which hybrids are intermediate and then manipulate a control group of organisms to quantify the effects of intermediacy on fitness in the field. This approach mirrors the way we think about EPI intuitively. In particular, it allows us to empirically estimate the shape of a fitness surface on which hybrids can be seen to fall in a valley between two peaks corresponding to the phenotypes of “pure” parental populations. Our results provide compelling evidence that extrinsic postzygotic isolation can provide a strong and primary barrier to gene flow at the early stages of ecological divergence.

## Results

We estimated the extent to which divergence in six important ecological traits (Figure 2) generates extrinsic postzygotic isolation among checkerspot populations adapted to feed on *Pedicularis semibarbata* and *Collinsis torreyi* (hereafter referred to as *Psem* and *Ctor*, respectively). We do so by comparing the traits and associated fitnesses of two classes of hybrid insects: hybrids between populations adapted to different host plants (different-host hybrids) and hybrids between populations adapted to the same plant (same-host hybrids). This comparison isolates the consequences of divergent host adaptation (affecting only different-host hybrids) from those of genetic drift and other types of local adaptation (affecting both different- and same-host hybrids) [34],[42],[43]. All insects used in our experiments were produced by crossing butterflies from four populations—two adapted to *Psem* and two adapted to *Ctor* (Figure 1C). Throughout this article, we refer to particular types of hybrids using two-letter symbols (PP, PC, CP, CC) where the first letter indicates the mother's traditional host and the second letter indicates the father's traditional host. We refer to “pure” insects stemming from crosses within a single population using one-letter symbols (P or C) indicating the population's traditional host. We address each trait and its fitness impacts, below, in the temporal order with which those impacts occur during the life cycle of a hybrid insect.

### Early Larval Performance Mediates Weak Asymmetric Isolation

We examined the performance of young hybrid larvae by placing hybrid eggs on naturally growing host plants in the field one day before hatching and then monitoring the growth and survival of the resulting larvae for 10 d. Hybrids were only examined on the host to which their mothers were adapted, and thus on which their mothers would have laid their eggs: CP and CC on *Ctor*, and PC and PP on *Psem*. Pure larvae were also included on their traditional hosts for reference: C on *Ctor* and P on *Psem*. Same-host hybrids did not differ from the corresponding pure larvae in either growth or survival (CC = C on *Ctor*, PP = P on *Psem*; Figure S1). Different-host hybrids performed well on *Ctor* (CP = CC/C for both growth and survival; Figure S1A, Table S1). However, on *Psem* they grew 15%–30% more slowly (PC<PP/P for growth; PC = PP/P for survival; Figure S1B, Table S2). The trend for reduced growth on *Psem* was significant only when predators were excluded (ANOVA *p* = 0.0001 and 0.18 in the absence and presence of predators, respectively; Table S2), probably because our predator exclusion technique allowed us to control for variation in the quality of individual *Psem* plants (see Methods). In summary, hybrids between populations adapted to *Psem* and *Ctor* were at a slight disadvantage on one of the two host plants. This weak, asymmetric effect is likely to be extrinsic since it disappeared on a third host (see section entitled “Different-Host Hybrids Are Not Intrinsically Unfit”).

### Intermediate Larval Foraging Height Mediates Moderate Isolation

#### Different-host hybrid larvae forage at intermediate heights

**Figure 3:**
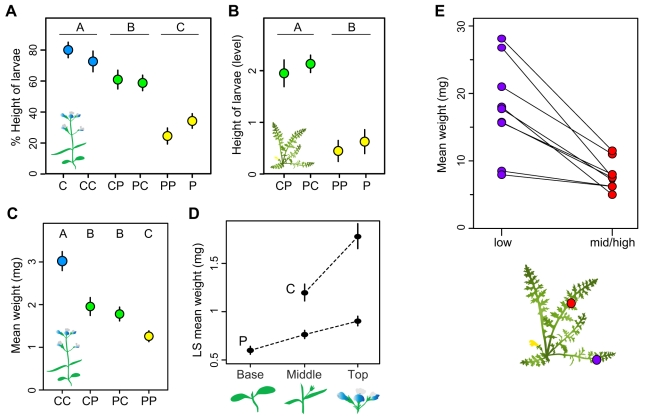
Different-host hybrid larvae forage at intermediate heights, slowing growth on both host plants. (A) Mean foraging height on *Ctor* plants (expressed as a percentage of the height of the plant). Different-host hybrids foraged lower than larvae with 2 *Ctor*-adapted parents (C/CC) but higher than larvae with 2 *Psem*-adapted parents (P/PP). Data analyzed using an ANOVA that examined differences among major groups (C/CC versus CP/PC versus P/PP) and among cross types within groups (C versus CC, CP versus PC, P versus PP). The former effect was significant (*p*<0.0001); the latter was not (*p* = 0.3). Circles show means ± SEM. Capital letters at top of panel show Tukey's HSD groupings. Data on pure types (C and P) were previously published in [40]. (B) Mean foraging height on *Psem* leaves. C and CC larvae were not included since they do not accept *Psem* as a host. Data analyzed as in (A). (C) Mean weight of hybrid larvae allowed to forage freely on mature *Ctor* plants for 10 d. Variation was significant (ANOVA *p*<0.0001; Table S3). Capital letters at top of panel indicate Tukey's HSD groupings. (D) Growth rates of pure larvae reared on basal, middle, and top sections of mature *Ctor* plants in small cups. Larvae grew more quickly when fed top sections than when fed middle/basal sections. LS means come from ANOVAs described in Table S4. All means were different from one another using Tukey's HSD. (E) Growth rates of pure P larvae placed on low versus mid-high leaves of naturally growing *Psem* plants in the field. Lines connect data from paired groups placed on the same plant. Larvae on lower leaves grew more rapidly than those placed on mid-high leaves (Table S5).

Different-host hybrid larvae foraged at intermediate heights relative to larvae with two parents adapted to the same host. On *Ctor*, the mean positions of first instar CP and PC larvae were slightly lower than those of CC and C larvae, and substantially higher than those of P and PP larvae (Figure 3A; ANOVA *p*<0.0001). The difference between CP/PC and P/PP was replicated when second instar larvae were tested on *Psem* (Figure 3B; ANOVA *p*<0.0001). On neither host did we observe a significant difference between reciprocal different-host hybrids (CP = PC) or between pure larvae and the corresponding same-host hybrids (C = CC, P = PP).

#### Intermediate foraging height slows growth on *Ctor*

The intermediate foraging heights of CP larvae on *Ctor* did not affect their fitness to the extent that we could detect a significant effect when they were placed on young, budding *Ctor* plants in the experiments described under “Early Larval Performance Mediates Weak Asymmetric Isolation” (Figure S1A). *Ctor* senesces from the bottom up, however, and we hypothesized that foraging height might affect fitness on more mature plants, whose lower leaves are senescing despite the presence of new growth at the top. Checkerspot larvae are often found on such plants in nature [44]–[48].

To test this, we first asked whether different-host hybrids do indeed suffer reduced fitness when placed on more mature, blooming to post-blooming *Ctor* plants. Growth rates varied as expected. CP and PC larvae grew more slowly than CC larvae but faster than PP larvae (Figure 3C; ANOVA *p*<0.0001; Table S3). Survival did not vary (ANOVA *p* = 0.7; Table S3). We then asked whether differences in foraging height might generate this growth effect due to variation in the nutritional quality of plant material found at different heights. We separated the base, middle, and top sections of blooming to post-blooming *Ctor* and fed them to pure C and P larvae in small cups. Both types of larvae grew faster on top plant sections, consisting of flowers, developing buds, and young leaves, than they did on middle sections, consisting of slightly older leaves (Figure 3D; ANOVA *p* = 0.0004 and 0.0017 for C and P larvae, respectively; Table S4). P larvae raised on the oldest leaves from the base of the plant grew the most slowly (Figure 3D). Survival did not vary (ANOVA *p* = 0.3; Table S4). These results indicate that intermediate foraging height puts different-host hybrids at a disadvantage on mature *Ctor* plants.

#### Intermediate foraging height slows growth on *Psem*

Intermediate foraging height did not contribute to the reduced growth rates of newly hatched PC larvae on *Psem* (described in the section on early larval performance) since all larvae remained at their natal site for the duration of our experiment (see “Natural History” section in Methods). However, foraging height may affect the fitness of older, late second and third instar larvae that stray from their natal site. We tested this by monitoring paired groups of larvae, one group placed low and one group placed middle to high on naturally growing *Psem* plants in the field. Larvae placed on mid to high leaves grew ∼50% more slowly than those placed on low leaves near the ground (Figure 3E; ANOVA *p* = 0.0002; Table S5). Whether larvae were placed on upper or lower leaves had no effect on survival (ANOVA *p* = 0.4; Table S5). In summary, intermediate foraging height slows larval growth on *Psem* as well as *Ctor*, putting different-host hybrids at a disadvantage and thereby generating moderate EPI.

### Intermediate Oviposition Behavior in Adult Females Mediates Strong Isolation

#### Different-host hybrid oviposition behaviors are intermediate

**Figure 4:**
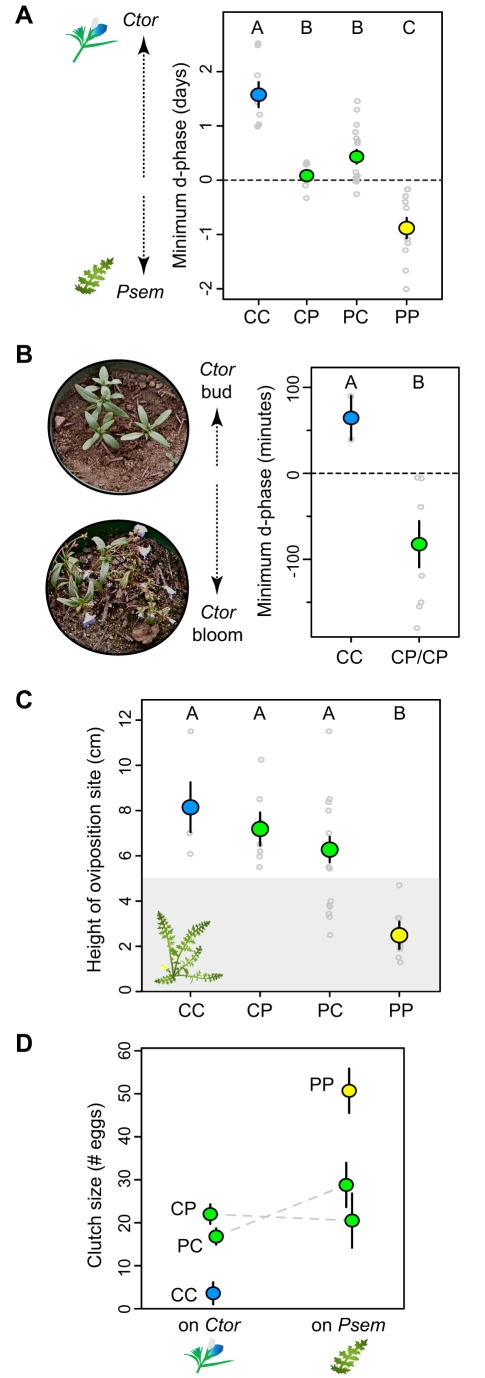
Different-host hybrid females exhibit intermediate oviposition behavior. Colored circles show means ± SEM and small grey circles show raw data. (A) Host Preference for *Ctor* versus *Psem*. Preferences are quantified in terms of the minimum discrimination phase (d-phase), whose absolute value and sign reflect preference strength and direction, respectively. The dashed line at zero indicates no preference. Variation among cross types was significant (ANOVA *p*<0.0001). Capital letters at top of panel show Tukey's HSD groupings. (B) Host preference for budding versus blooming *Ctor*. Positive and negative d-phases indicate preferences for budding and blooming plants, respectively. Typical examples of such plants are shown in the photographs. The difference between CC and CP/PC was significant (*t* test *p* = 0.03) and is reflected by capital letters at top of panel. (C) Oviposition site height on *Psem*. All females were placed on leaves above 5 cm (white portion of plot); females choosing oviposition sites below 5 cm (grey portion of plot) dropped towards the ground during their exploration of the plant. Variation among cross types was significant (ANOVA *p*<0.0001). Capital letters at top of panel show Tukey's HSD groupings. (D) Clutch size. Variation was significant with [PC = CP]>CC on *Ctor* (ANOVA *p*<0.0001) and [PC = CP]<PP on *Psem* (ANOVA *p* = 0.003).

We quantified the host species preference of ovipositing adult hybrids using a continuous variable called the discrimination phase or d-phase, measured in units of time (see Methods for more detail). Same-host hybrid females expressed preferences that were indistinguishable from those of their pure counterparts, reported elsewhere [40]; PP females moderately preferred *Psem* (mean d-phase = −0.7 d), and CC females strongly preferred *Ctor* (mean d-phase = +1.2 d). Different-host hybrids were intermediate, differing significantly from both same-host hybrid classes (Figure 4A; ANOVA *p*<0.0001). They readily accepted both hosts and showed little to no preference (CP mean d-phase = +36±78 min, PC mean = +182±55 min). The marginally significant tendency for PC females to be more *Ctor* preferring than CP females (ANOVA contrast *p* = 0.14, *t* test *p* = 0.03) is suggestive of sex linkage at loci of small effect, since females are the heterogametic sex in butterflies and inherit their only Z chromosome from their fathers.

We also tested hybrid female oviposition preferences for *Ctor* plants of varying phenological stage. Although we were only able to test a few same-host hybrid individuals, CC females appeared to share the tendencies of pure *Ctor*-adapted butterflies; all 3 CC females tested preferred budding *Ctor* over blooming *Ctor* (Figure 4B). Interestingly, different-host hybrids were more similar to *Psem*-adapted females; all 8 females tested (3 CP and 5 PC) preferred blooming plants (Figure 4B). This difference (CC versus CP/PC) was significant both in terms of the proportion of butterflies with one or the other preference (FET *p* = 0.0006) and in terms of quantitative d-phases (*t* test *p* = 0.03).

We tested hybrid female oviposition site height on *Psem* only since *Ctor* does not offer many oviposition site options near the ground (Figure 1A). PP females resembled their parents, showing a strong innate tendency to drop to the base of the plant and lay their eggs within a few cm of the ground (Video S1; Figure 4C; mean height = 2.5±0.6 cm). While a few different-host hybrids seemed to share this phenotype, most neglected to explore the plant and chose oviposition sites near the point where they were first placed (Video S2; Figure 4C; PC mean height = 6.2±0.6, CP mean height = 7.2±0.7). The few CC females that accepted *Psem* also neglected to explore the plant and laid their eggs well above 5 cm (Figure 4C). Overall, variation among hybrid types was significant (ANOVA *p*<0.0001), with different-host hybrid females placing their eggs significantly higher than PP insects and slightly, but insignificantly, lower that CC insects.

We assayed hybrid clutch sizes by counting the number of eggs they laid per clutch on their preferred host species (or both host species for different-host hybrids). Same-host hybrids laid the same number of eggs per clutch as their pure counterparts, reported elsewhere (Figure 4D; CC mean = 4±3 eggs, PP mean = 51±5) [40]. Different-host hybrids laid intermediate sized clutches on both host plants. They laid significantly larger clutches on *Ctor* than CC females (ANOVA *p*<0.0001; CP mean = 22±2, PC mean = 17±2) and significantly smaller clutches on *Psem* than PP females (ANOVA *p* = 0.003; CP mean = 21±6, PC mean = 29±5) (Figure 4D).

In summary, different-host hybrid butterflies exhibited oviposition behaviors that were either intermediate to those of same-host hybrids (preference for host species, oviposition site height, clutch size) or at least significantly different from the same-host hybrid type whose behavior was relevant on the given host (preference for individual *Ctor* plants). In the following sections, we describe experiments designed to estimate the effects of these behaviors on offspring fitness.

#### Intermediate oviposition behaviors reduce offspring fitness on *Ctor*

**Figure 5:**
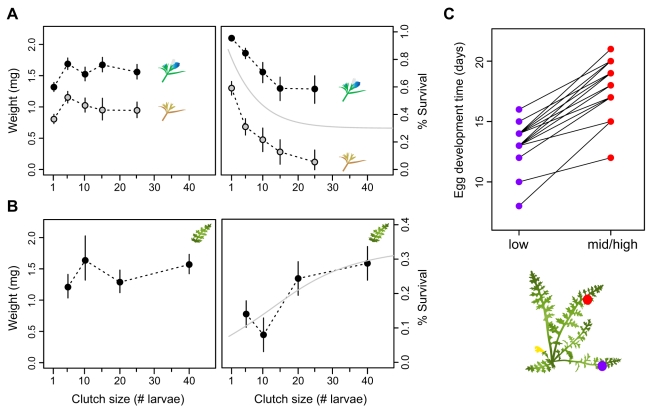
Intermediate oviposition behavior reduces offspring fitness. (A) Effects of clutch size and host phenology on *Ctor*. LS mean weights and survival ± SEM of neonate larvae left on blooming plants (black circles) and senescing plants (grey circles) in groups of various size for 10 d. Both growth and survival were higher on the blooming plants than on the senescing plants. Survival also decreased with increasing group size. LS means come from ANOVAs described in Table S6. The grey line in the right-hand panel represents a monotone cubic spline fit to the combined data from blooming and senescing plants. (B) Effects of clutch size on *Psem*: LS mean weights and survival ± SEM of neonate larvae left on plants in groups of various size for ∼10 d. Larvae in larger groups survived better than those in smaller groups. LS means come from ANOVAs described in Table S7. The grey line in the right-hand panel represents a monotone cubic spline fit to the data. (C) Effects of oviposition site height on egg development time on *Psem*: Each line connects data from low and mid/high clutches laid on the same plant. Eggs on low leaves developed more rapidly than those on mid/high leaves (Table S8).

Different-host hybrid females ovipositing on *Ctor* chose to lay larger clutches on phenologically older plants than *Ctor*-adapted butterflies. To estimate the effects of this behavior on offspring fitness, we examined the performance of newly hatched larvae placed in small to medium-sized groups on blooming versus senescing plants in the field. Note that the lifespan of *Ctor* is short, such that placing neonate larvae on blooming and senescing plants simulates the experience of larvae hatching from eggs laid 10–14 d earlier on plants that were budding and blooming, respectively (see Methods). Single larvae grew slightly more slowly than those placed in groups of 5, 10, 15, and 25, but there was no clear overarching relationship between clutch size and growth rate (Figure 5A, left-hand panel; ANOVA *p* = 0.001; Table S6). In terms of survival, however, single larvae performed significantly better than the other group sizes. There was a clear trend for decreasing survival with increasing clutch size on both blooming and senescing plants (Figure 5A, right-hand panel; ANOVA *p*<0.0001; Table S6). As expected in light of previous field observations and experiments [44]–[47], larvae placed on senescing plants suffered serious reductions in both growth and survival in comparison to those placed on blooming plants (Figure 5A, both panels, compare black and grey circles). They grew ∼28% more slowly and were ∼70% less likely to survive to 10 d of age (ANOVA *p*<0.0001 for both response variables; Table S6). We did not detect an interaction effect between clutch size and plant phenology on either growth or survival (*p*>0.3). In summary, the oviposition behavior of different-host hybrids is doubly maladaptive on *Ctor*. Medium-sized clutches reduce offspring survival, and choosing to lay on phenologically advanced plants reduces both offspring survival and growth.

#### Intermediate oviposition behaviors reduce offspring fitness on *Psem*

Different-host hybrids ovipositing on *Psem* laid medium-sized clutches relative to the large clutches laid by *Psem*-adapted butterflies. We estimated the fitness effects of this behavior by examining the growth and survival of newly hatched larvae put out in medium to large-sized groups on naturally growing plants in the field. Clutch size had a significant effect on survival, but not weight (Figure 5B; ANOVA *p* survival = 0.01, *p* weight = 0.4; Table S7). More specifically, survival tended to increase with increasing clutch size (Figure 5B, right panel), a relationship opposite to that observed on *Ctor* (Figure 5A, right panel).

Most different-host hybrids ovipositing on *Psem* also differed from *Psem*-adapted butterflies in failing to drop to the bottom of their host to place their eggs near the ground. The data presented in Figure 3E indicate that this failure would significantly slow offspring growth during the period of time when larvae remain at the natal site (10–14 days or 1–2 instars). To test for parallel effects on egg development time, we manipulated pure *Psem*-adapted females into laying paired clutches, one high and one low, on naturally growing *Psem* plants in the field and recorded the time to hatching. Eggs laid on middle to upper leaves took ∼27% longer to hatch than those laid within a few centimeters of the ground (Figure 5C; ANOVA *p*<0.0001; Table S8). In summary, the oviposition behavior of different host hybrid females is maladaptive on *Psem*, just as it was on *Ctor*, further enhancing EPI. Medium clutch sizes reduce offspring survival and high oviposition sites slow offspring development.

### Different-Host Hybrids Are Not Intrinsically Unfit

The nature of the behavioral phenotypes addressed in this article makes it clear that their effects on fitness are extrinsic rather than intrinsic. This distinction is not as clear, however, with regard to the reduced growth rates of young PC larvae on *Psem* (see first section of Results). We therefore examined larval growth rate on a third host plant, *Castilleja applegatei*. If the original reduction in growth on *Psem* is extrinsic, it may dissipate on this “neutral” host [12]. Indeed, PC larvae grew just as quickly as P and PP larvae when reared on *C. applegatei* (*p* = 0.11; Figure S2A). The neutral environment test is not fail-safe since intrinsic fitness problems may also fail to penetrate in certain environments ([6]: p. 250). We therefore searched for other signs of inherent incompatibilities in different-host hybrids by examining egg viability, female teneral weight, sex ratios, and fertility. We found none (Figure S2).

### Visualizing EPI Using an Adaptive Surface

**Figure 6:**
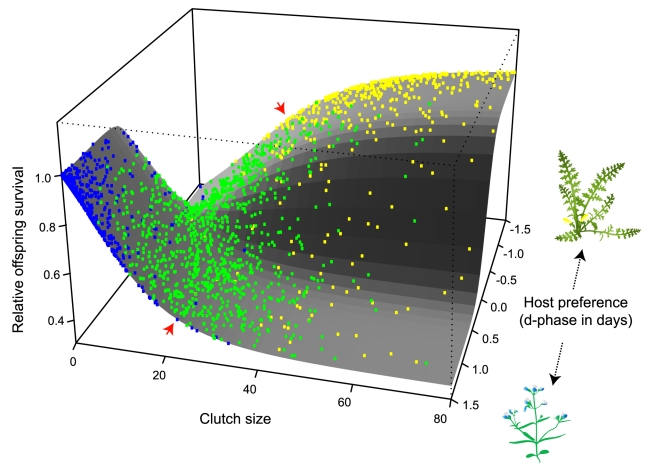
Divergence in host preference and clutch size among *Psem*- and *Ctor*-adapted populations generates strong extrinsic postzygotic isolation. Each dot represents a hypothetical CC (blue), CP/PC (green), or PP (yellow) female. Different-host hybrids fall in an adaptive valley between the two peaks occupied by the two types of same-host hybrids, signaling the presence of significant EPI. The phenotypes of these hypothetical females were drawn from the distributions that best fit our observed data (see Methods). The scatter of the dots thus represents the biological variation and experimental noise present in our data. The shape of the surface was estimated based on field experiments. The small red arrows point to the curved edges of the surface that illustrate the relationship between offspring survival and clutch size on *Ctor* (foreground curve) and *Psem* (background curve). These curves were estimated directly from the data shown in Figure 5A and 5B and are represented in that figure as light grey lines. The front to back axis represents host preference. Thus, for females with large intrinsic clutch sizes (right-hand side of the plot), expected offspring survival goes from being relatively low for females that are most likely to lay on *Ctor* (foreground = positive d-phases) to relatively high for females that are most likely to lay on *Psem* (background = negative d-phases).

Extrinsic postzygotic isolation is often conceptualized using the metaphor of an adaptive surface; it occurs when hybrids are intermediate and fall in an adaptive valley between two fitness peaks occupied by the parental populations. We transformed our empirical data on hybrid host preferences and clutch sizes into such a surface in order to help visualize the extent to which intermediate values of these traits generate isolation. Other phenotypes were not included since doing so would require more than three dimensions. The shape of the surface was estimated by combining our field data on the survival of various sized clutches (Figure 5A and 5B, right panels) with a logical translation of host preference values into probabilities of laying on each host (see Methods). The surface has two peaks corresponding to the optimal clutch size on each host plant (Figure 6). The position of same and different-host hybrids on the surface was simulated based on the results of our host preference and clutch size assays (Figure 4A and 4D; see Methods). The two types of same-host hybrid females resemble pure insects and sit upon the two peaks; CC females strongly prefer *Ctor* and lay small clutches (Figure 6, blue dots), while PP females prefer *Psem* and lay large clutches (Figure 6, yellow dots). Different-host hybrids, on the other hand, fall in the adaptive valley between the peaks; they readily accept both host species and lay their eggs in medium-sized clutches (Figure 6, green dots).

## Discussion

The idea that extrinsic postzygotic isolation can create a strong barrier to gene flow at the early stages of ecological divergence and play a critical role in speciation is relatively unsubstantiated. Examples of this type of reproductive isolation are few and tend to involve taxa at the intermediate to advanced stages of the speciation process—forms or sister species that are also separated by substantial mating barriers and have already proven their ability to coexist in sympatry or parapatry. Moreover, EPI has not been found in several systems where the magnitude of ecological divergence suggests that it should be strong (e.g., [49],[50]).

In this study, we searched for EPI among allopatric populations of a single butterfly species that have adapted to different host plants. Rather than examining the overall fitness of hybrid insects, we attempted to tease apart potential underlying ecological incompatibilities one by one. For traits known to have diverged between the parental host-adapted populations, we first asked whether different-host hybrids were intermediate, and second whether intermediate phenotypes reduced growth and survival in the insects' natural field setting. This approach has the advantage of cleanly dissociating the fitness effects of intermediate hybrid traits from those of inherent incompatibilities and provides a mechanistic understanding of EPI that mirrors the way we think about the phenomenon intuitively. Our results demonstrate that different-host hybrids lack intrinsic developmental defects but display intermediate phenotypes that are maladaptive on both hosts in nature, resulting in substantial extrinsic isolation.

### Mechanisms of EPI Among *E. editha* Populations That Specialize on *Ctor* and *Psem*

Among the traits we examined in different-host hybrid insects, oviposition behaviors were the most maladaptive. First, medium clutch sizes lowered offspring survival on both hosts—with small clutches being optimal on *Ctor* and large clutches being optimal on *Psem*. This effect is distilled in Figure 6. Interestingly, predator pressure is substantially higher on *Psem* than on *Ctor* (compare mean survival of all larvae on two hosts in Figure S1; [46]) and unpublished experiments indicate that the advantage enjoyed by large clutches on *Psem* disappears when predators are excluded. Host-specific predator regimes may therefore help explain why the relationship between clutch size and offspring survival was opposite on the two hosts.

Different-host hybrid females accrue further fitness losses due to additional maladaptive oviposition behaviors. When laying on *Ctor*, they preferred phenologically older plants on which offspring fitness was severely compromised (30% growth deficit, 70% survival deficit). It makes sense that a preference for older plants would be maladaptive on an annual plant like *Ctor*. Coping with early host senescence is a major challenge for *E. editha* populations that use annual hosts [44],[45],[47],[48]. Larvae cannot enter diapause until mid to late 3^rd^ instar, yet many find themselves on senescing plants as 1^st^ or 2^nd^ instars (28% of 53 clutches censused in 1995 and 32% of 25 clutches censused in 2009 at TR). When laying on *Psem*, different-host hybrids differed from *Psem*-adapted insects in failing to lay their eggs near the ground. Instead, they laid at the place where they first contacted and tasted the plant (compare Videos S1 and S2). Field experiments again revealed that this behavior is maladaptive, reducing offspring development time by 30%–50%. Preliminary thermal data suggest that eggs and larvae found near the ground develop more quickly than those found on middle to upper *Psem* leaves because they are closer to the hot, sandy soil and therefore experience a warmer microenvironment.

Although the behavior of hybrid larvae was less deleterious than that of adult females, it affected both sexes and is also expected to contribute to isolation. Different-host hybrid larvae foraged at lower levels on *Ctor* than insects with two *Ctor*-adapted parents, which foraged at the top. This behavior did not have a detectable fitness effect when larvae hatched onto young, still budding *Ctor* plants but probably contributed to the 15% growth deficit observed when larvae were reared on mature plants. *Ctor* senesces from the bottom up, and plant material found at the base and middle of the plant was less nutritious than that found at the top. *Psem*, on the other hand, is a perennial herb that retains moisture throughout the summer. Host senescence is not a problem for larvae on this host. Moreover, the youngest leaves appear in the center of the plant, being neither high nor low (Figure 1A). These differences, in combination with the potential temperature effect mentioned above, help explain why the relationship between foraging height and growth rate on *Psem* was opposite to that on *Ctor*.

The last trait we show to contribute to isolation among *E. editha* populations adapted to *Ctor* and *Psem* is early larval performance. However, the effect was asymmetric and relatively weak, consisting of a 15%–30% growth deficit on *Psem* only. We suspect that different-host hybrids have problems digesting or detoxifying *Psem*, but could not test this directly due to the difficulty of manipulating an insect's physiology independently from other traits. We can at least rule out the contribution of foraging height since the larvae in our experiments on *Psem* did not stray from their natal site. It is also unlikely that intrinsic incompatibilities contributed, since different-host hybrids showed no signs of any developmental defects and grew just as quickly as PP insects on both *Castilleja applegatei* and young *Ctor*.

### An Overall Estimate of EPI in *E. editha*?

Although we could attempt to synthesize the various growth and survival deficits described above into a single measure of overall hybrid fitness, this exercise would give the false impression that our understanding of the system is complete. For one, although we expect *E. editha* growth rates to correlate with lifetime fitness, the exact relationship between growth, survival, and reproduction is poorly defined. Second, there may be other relevant trait differences between populations of which we are unaware—for example, in males or post-diapause larvae. Lastly, our estimates come from a single year and do not incorporate annual environmental fluctuations likely to influence the overall strength of EPI (e.g., [16],[51]).

Despite these caveats, the diversity of traits that we show to affect hybrids and the surprising strength of their effects in at least one place and time make it clear that extrinsic postzygotic isolation provides a substantial overall barrier to gene flow in this system. We found not a single positive effect of hybridization on fitness. Neither do we expect that effects associated with one trait might mitigate those stemming from another. Instead, the deleterious effects are likely to accumulate over the life cycle. As an example of how this might occur, consider the hybrid offspring of an adult male *E. editha* that immigrated from a *Psem*-adapted population to a *Ctor*-adapted population and mated with a local female. Eggs would be laid on *Ctor*, the plant preferred by the female. Hatching larvae would forage lower on the host than local larvae and, in most years, would grow more slowly due to their failure to locate the best food at the host meristems. This reduction in growth rate would increase larval mortality due to host senescence and extend the period of exposure to parasitoid attack. Those hybrids that survived to adulthood would be smaller and/or eclose later than local *Ctor*-adapted butterflies—with a penalty paid in fecundity and/or time. Adult female hybrids would have weak host preferences, sometimes choosing to lay eggs on *Psem* and sometimes choosing *Ctor*. When laying on *Psem* they would lay clutches too small for optimal survival on that host and oviposit high enough on the plant to slow the development of their eggs and young larvae. When laying on *Ctor*, they would tend to choose the least suitable, most phenologically advanced individuals and then lay clutches too large for optimal survival on that host. Note that the majority of these fitness costs stem from behaviors specific to females. EPI should therefore be most effective at blocking the flow of maternally inherited genes, including those found on the W chromosome and in the mitochondrial genome.

We cannot rule out the possibility that different-host hybrids would perform well on a host plant that we have not investigated, for example, one of the host plants used by *E. editha* in other parts of its range. However, no other known *E. editha* host plants are associated with butterflies showing the unique combination of phenotypes that characterize the different-host hybrids studied here. Moreover, work from other systems suggests that colonization of a novel environment by hybrid organisms sometimes generates a third species, rather than impeding or reversing divergence between the two parent species [52]–[55].

### The Importance of Field-Based Studies

Although related, *Ctor* and *Psem* differ remarkably in chemistry [40], growth form (Figure 1A), life history (annual versus perennial), and visitation rate by *E. editha* predators [46]. As discussed above, each of these is likely to play a role in mediating the fitness costs of the traits addressed in this study. Several of them, however, are difficult or impossible to replicate indoors, and we would have underestimated the strength of EPI had we conducted our experiments in the laboratory or greenhouse. For example, the predators that put smaller clutches at a disadvantage on *Psem* would not have been present in the laboratory. Even field cages designed to prevent the movement of larvae on naturally growing plants would have excluded predators and thus been problematic. Likewise, the temperature gradient we suspect of slowing the development of eggs and larvae found on the upper leaves of *Psem* plants would have been difficult to duplicate in the laboratory. This study thus highlights the importance of estimating EPI in the field when possible.

Most previous studies of extrinsic postzygotic isolation in insects that specialize on distinct host plants were conducted in the laboratory ([20],[23],[25],[27],[30], but see [21]). These studies revealed growth and survival deficits in F1 individuals ranging from 10%–50% (averaged across both host plants; Table S9). We suspect, however, that EPI may be significantly stronger and more prevalent than the lab estimates suggest. Indeed, most existing estimates of EPI, including our own, are best interpreted as minima, either because they come from the laboratory or because they incorporate only a subset of life stages and traits.

### Behavioral Preferences as Widespread Drivers of Extrinsic Postzygotic Isolation

By focusing on specific traits, we have shown that behavioral divergence is a potent source of ecological selection against hybrids in this system. There are a few additional examples of this kind. One example involves apple and hawthorn host-races of the apple maggot-fly *Rhagoletis pomonella*. These insects orient towards the odor of their own host and avoid the odor of the alternative host. F1 hybrids avoid the odors of both parental hosts and are likely to have problems finding suitable oviposition sites in nature [32],[56]. Other examples come from bird populations that use different wintering grounds, or that migrate along different routes in order to avoid geographic barriers on their way to the same wintering grounds [33],[57],[58]. In at least one case, hybrids between SE- and SW-migrating European blackcap populations inherited a genetic tendency to migrate in an intermediate southerly direction—a behavior expected to take them directly over formidable geographic barriers including the Alps, the Mediterranean Sea, and the Sahara desert [33]. Both of these examples involve the inheritance of intermediate niche preferences—with the niches taking the form of host plants in the first example and wintering grounds in the second. One of the most deleterious behavioral incompatibilities described in the current study also involved niche preference (for phenologically unsuitable *Ctor* individuals). The fact that adaptation to a new niche is often accompanied by the evolution of a new preference for that niche suggests that maladaptive behavioral preferences may be widespread drivers of EPI in animals.

### Strong EPI in the Absence of Direct Links between Niche-Adaptation and Mate Choice

As outlined in the introduction, the best-documented ways in which ecological selection leads to reproductive isolation involve mating barriers. These barriers arise when niche-adaptation is directly linked to mate choice. For example, many insects choose mates from among those they meet on their preferred host plant, causing habitat isolation (e.g., [37],[59]–[62]). Many plants can only exchange pollen with those whose flowers attract the same suite of pollinators, causing pollinator isolation (e.g., [63],[64]). In some organisms, ecological traits are used as, or affect, mating cues, causing sexual isolation (e.g., [35],[65],[66]).

Interestingly, ecological mating barriers appear to be absent in this system. *E. editha* does not mate on its host on a micro-scale, and the habitats occupied by populations adapted to *Psem* and *Ctor* are essentially the same on a macro-scale. This means that neither immigrant inviability nor habitat isolation should prevent local insects from mating with differently adapted migrants. Furthermore, the growing seasons of the two host plants both begin at snow melt in early spring, leaving little opportunity for temporal isolation between the two types of populations. At first glance, the absence of such host-associated mating barriers may explain why *E. editha* host shifts are not generally associated with speciation events [67]–[69]. Nevertheless, consideration of the particular host plants and populations addressed here tells a complementary story. *Psem* and *Ctor* (and their respective genera) are unique among hosts of *E. editha* in the degree and dimensionality of divergent adaptation that is associated with feeding on them [40]. Perhaps as a result, the two plants are never used jointly by a single generalized population. Instead the populations that use them remain stubbornly allopatric, despite widespread host sympatry.

Our finding of substantial EPI in the absence of host-associated mating barriers provides an explanation for this curious geographic scenario and suggests a potential route to complete speciation. Imagine a mated female migrant from a *Psem*-adapted population arriving at a site where local butterflies use *Ctor* (or vice versa). She will likely lay eggs on her preferred host plant, *Psem*. The apparent absence of premating barriers, however, will make it difficult for her offspring to establish a stable, sympatric, host race. Instead, they are expected to mate with local insects and produce hybrids. Strong EPI, in turn, should stem the flow of genes from those hybrids back into the local population. The combination of little to no prezygotic isolation and strong EPI may thus explain the existence of a set of allopatric populations that are significantly isolated yet unable to move into sympatry. If this situation persists, non-ecological forms of prezygotic isolation may have time to accumulate, or increased migration rates may trigger reinforcement [70].

Whatever the ultimate fate of these populations, this study illustrates how extrinsic postzygotic isolation can accumulate over the life cycle of an insect and reduce gene flow between populations that are adapting to distinct ecological niches.

## Methods

### Natural History

In the montane populations studied here, *E. editha* completes a single generation per year. Adults fly anytime between May and July. Females lay eggs in clutches, which hatch after approximately 2 wk. Young larvae must feed for a further 2 wk, until mid-third instar, before they are able to enter diapause, which lasts through winter to snowmelt the following spring. Post-diapause larvae then resume feeding for 2–3 wk, pupate in the ground litter, and eventually eclose as adults.

*E. editha* is most specialized on particular hosts during the adult female and pre-diapause larval stages. Adult females are adapted to recognize their host species and lay a particular-sized clutch at a particular height upon it (Figure 2) [40]. Pre-diapause larvae are adapted to digest/detoxify the host that their mother chose for them (Figure 2) [40]. They tend to remain with their siblings and commence feeding in the exact spot where they hatched until they run out of edible leaf material in the immediate vicinity. This means that larval position is determined by the mother's oviposition site choice for the first 2–4 d on *Ctor* (which has small, quickly defoliated leaves) and 10–14 d on *Psem* (which has larger leaves). After this time, pre-diapause larvae make their own decisions about where to feed and rest on the natal plant and are adapted to do so at particular heights (Figure 2) [40]. They generally stay on the natal plant until it is completely defoliated (usually at least 10 d). Post-diapause larvae are less specialized. For example, in populations where eggs and pre-diapause larvae are found only on *Psem*, wandering post-diapause larvae may feed extensively on newly germinated *Ctor* seedlings in early spring [71]. Pupae and adult males have no particular relationship with host plants.

### Matings and Experimental Insects

We mated butterflies from the four populations highlighted in Figure 1B according to the bold arrow combinations illustrated in Figure 1C. The females used in these matings were collected as pupae or final/penultimate instar larvae in the early spring of 2004 and 2005, reared to pupation on *Ctor* leaves in small cups, allowed to eclose in small cages, and kept in sealed plastic containers on ice for 0–7 d prior to mating. The males were caught as adults in their native habitats. All mating configurations were equally easy to achieve.

To obtain immature stages for our experiments, we solicited eggs from mated females in captivity. Hybrid eggs (CC, CP, PC, PP) were obtained from females mated as described above, while “pure” eggs (C and P) were obtained from wild-caught females that had mated with males from their own populations prior to capture. We incubated all eggs in the presence of leafy material from both host plants to control for the possibility that host-adaptive phenotypes may be induced by exposure of eggs to volatile plant chemicals.

To obtain adults for our oviposition behavior assays, we set aside 20–30 eggs from each of the females mated as described above and reared them to adulthood on *Ctor*. *Ctor* was used for all larvae, regardless of hybrid type, for three reasons: (1) *Ctor* is abundant and easy to gather/transplant, (2) all larvae develop faster on *Ctor* than on *Psem*, and (3) feeding all larvae the same host controls for larval host effects. Upon hatching, young larvae were reared to diapause on live or freshly gathered *Ctor*, and kept at 0–4°C through the winter in the basement room of an unheated cabin at Sagehen Creek Field Station (6,380 ft elevation in the Sierra Nevada of California). Buffered from above-ground fluctuations, the temperature conditions in this room mimic those experienced by naturally occurring larvae that spend the winter at similar elevations in the ground litter buried under many feet of insulating snow. In the subsequent spring (2006), the larvae were reared on live, potted *Ctor* under a shade cloth in the field. Those individuals that developed through to pupation were allowed to eclose in small cages and were weighed on a microbalance. Adult males were then housed in cages hanging in the shade and fed artificial nectar (honey, raw sugar, salt, and amino acids) once a day and offered mud puddles every 2–4 d. Adult females were placed in sealed plastic containers on ice for 0–21 d until we were ready to mate them and test their behavior. We mated most same-host hybrid females to different-host hybrid males and vice versa. More than 1–2 wk on ice prior to mating caused some females (regardless of cross type) to have difficulty laying eggs and/or to lay unhealthy, shriveled eggs. We excluded such females from our assays.

### Larval Performance and Foraging Height

#### Early larval performance

To examine larval performance on *Ctor*, we put out 2 clutches from each of 12 C families, 7 CC families, and 9 CP families in a single study area with consistent aspect and shade level. Each clutch comprised 4–6 eggs (typical clutch size for a *Ctor*-adapted mother) laid in captivity on a small piece of host material, referred to as an egg matrix. One day prior to hatching, we wedged the matrices among the branching stems at the top of *Ctor* plants growing in the study area (one clutch per plant), left them for 10 d fully exposed to predators and natural elements, and then returned to count and weigh remaining larvae. We are confident that the number of remaining larvae reflected their survival, because young *E. editha* larvae from our study populations are sedentary and generally do not leave the natal plant until it is defoliated. We used an ANOVA to examine the effect of cross type (C versus CC versus CP) on log-transformed weight and arcsine-transformed survival.

To examine larval performance on *Psem*, we conducted two experiments. The first was analogous to that on *Ctor* (described above) except that it involved P, PP, and PC families (*n* = 25, 23, and 27, respectively), clutches comprised 25–30 eggs (typical clutch size for a *Psem*-adapted mother), and Super Glue was used to affix each egg matrix to the leaf of a *Psem* plant (with care to avoid contact between the glue and the eggs themselves). Glue was necessary because, unlike *Ctor*, *Psem* is frequented by ants that quickly remove loose matrices. The study area configuration also differed from the *Ctor* experiment. Since *Psem* does not grow as densely as *Ctor*, we could not put out all clutches at a single site. Instead, we put out 12 clutches (4 P+4 PP+4 PC) at each of 14 sites (15 m×15 m areas with consistent aspect and shade level). To take this site effect into account during analysis, we saved the residuals from an ANOVA testing the effect of site on survival/weight (log/arcsine transformed) and then averaged these residuals across the two replicate clutches within each family before finally testing for an effect of cross type (P versus PP versus PC) on the resulting family averages. The low survival characteristic of larvae on *Psem* rendered several sites severely imbalanced with respect to the types of clutches for which we could obtain weight measurements at the end of the experiment. We excluded the three most severely imbalanced blocks (with <half of the clutches remaining) when analyzing larval weight.

In the second experiment on *Psem*, we excluded small, crawling predators using a sticky substance, Tanglefoot. We encircled the base of three leaves from each of 28 plants (two plants at each of the same 14 sites described in the first experiment) with a sheath of masking tape and smeared the Tanglefoot on top of the masking tape. The tape prevented the Tanglefoot from eroding the petiole, and the Tanglefoot prevented small, crawling predators from reaching larvae on the leaf. The Tanglefoot also prevented larvae from moving between leaves. One of the three leaves received 25 neonate P larvae, another received 25 PP larvae, and the last received 25 PC larvae. We left the larvae in the field for 9–13 d before returning to count and weigh the survivors. All larvae at a single site were left in the field for the same period of time. We used an ANOVA to examine the effects of site (14 sites), plant nested within site (two plants per site), and cross type on log transformed weight and arcsine transformed survival. The inclusion of the plant effect in this analysis allowed us to control for variation in the quality/microclimate of individual *Psem* plants when comparing the fitness of P, PP, and PC larvae—something we could not do in the first experiment since each clutch was placed on a different plant.

#### Larval foraging height

We examined larval foraging height on *Ctor* by placing groups of 10 neonate larvae on potted plants set out under a shade cloth in the field, allowing them 2–4 d to establish themselves, and then recording their position as a fraction of the height of the plant. We tested the following number of families for each cross type: C = 16, CC = 9, CP = 11, PC = 16, PP = 15, and P = 18. In an analogous experiment on *Psem*, we placed groups of five second instar larvae on a single *Psem* leaf with three tiers of leaflets standing upright in an Eppendorf tube of water. We then recorded, several times over the course of 2–3 d, whether the larvae were on the ground (level 0) or on the first, second, or third tier of leaflets (levels 1, 2, and 3). We tested the following number of families for each cross type: P = 10, PP = 13, PC = 19, and CP = 8. C and CC families were not included because they do not readily accept *Psem* as a host. We used ANOVAs to examine the effect of cross type on mean larval position (averaged across siblings and observations within families) for both experiments.

#### Fitness effects of larval foraging height on Ctor

We reared larvae in small cups on the basal, middle, and top parts of blooming to post-blooming *Ctor* plants. The basal parts included the lowest part of the stem carrying the cotyledons and the lowest pair of leaves. The middle parts included the middle section of the stem carrying the next youngest 1–2 pairs of leaves. The top parts included the young, developing leaves, buds, and flowers from the apex of the plant. In each of 15 replicates, we divided a single plant into its base, middle, and top, and fed each of the three parts to a separate group of five neonate P larvae from the RM population. Larvae were fed in small cups for ∼10 d and were not allowed to run out of food. We did the same with neonate C larvae from TR except that we included middles and tops only, excluding bases. We analyzed the two types of larvae separately in ANOVAs that tested for the effects of plant (15 replicates) and level (base versus middle versus top for P larvae; middle versus top for C larvae) on log transformed weight and arcsine transformed survival. Plants for which survival on one or more parts was zero were rendered imbalanced with respect to weight measurements and were therefore excluded from the weight analysis. We conducted a second experiment to determine whether these growth effects have the potential to translate into a fitness cost to freely roaming larvae that forage at different heights on upright plants. Groups of 10 neonate larvae from same- and different-host hybrid families were left to forage on potted blooming to post-blooming *Ctor* plants set out under a shade cloth in the field. We tested the following number of families: CC = 16, CP = 15, PC = 28, and PP = 23. We recorded larval weight and survival after 10 d and tested for variation among hybrid types using one-way ANOVAs.

#### Fitness effects of larval foraging height on Psem

To examine the fitness effects of larval foraging height on *Psem*, we collected naturally laid P eggs from the field at RM, divided them into 36 groups of 20 eggs each, and allowed each group to hatch, feed, and begin spinning a web on a freshly cut *Psem* leaf in a small cup for 24 h. We then removed the leaves (and the larvae they carried) from the cups and tied them with dental floss to one low leaf and one high leaf from each of 18 naturally growing *Psem* plants. We left them in the field for 10 d, or until either clutch from a given plant ran out of food on the leaf to which it was tied, before returning to count and weigh the survivors. The opportunity to begin feeding and spinning a web together for 24 h, and the fact that we did not allow them to run out of food, ensured that the larvae stayed where they were first placed on the plant. We used an ANOVA to estimate the effects of plant (replicate) and level (low versus high) on log/arcsine-transformed growth/survival. Plants for which survival in either the low or high clutch was zero were imbalanced with respect to weight measurements and were excluded from the weight analysis.

### Adult Female Oviposition Behavior and Their Effects on Fitness

#### Host preference

We tested the oviposition preferences of female butterflies using a bioassay in which we recorded their responses to repeated encounters staged with each host in alternation. This testing technique has been described in detail and its assumptions tested by [72] and [73]. The goal is to estimate the strength of a female's preference for one host over the other by determining how long she would continue searching unsuccessfully for her preferred host before becoming willing to accept the less preferred host upon encountering it. In an analogy to humans, one can imagine trying to estimate the strength of a human's preference for chocolate over carrots in the following way. We offer a woman the two items in alternation, without allowing actual consumption. We record the time at which she first expresses a willingness to eat the chocolate (“accepts” the chocolate). We then remove the chocolate (before she eats it) and continue to offer the carrot every 30 min and record the time at which she finally becomes hungry enough to accept that. The length of time that passes from first acceptance of the chocolate to first acceptance of the carrot provides a quantitative measure of preference—the longer the time, the stronger the preference. We call this period of time the discrimination phase (d-phase). It is not necessary to know a priori that the woman prefers the chocolate. If she had preferred the carrot, she would have accepted it first, and we would have then continued by offering the chocolate every 30 min until it was also accepted.

We used this approach with butterflies, offering a female each host in alternation and recording her response, without allowing oviposition. We define acceptance as settling on the host, curling the abdomen, extruding the ovipositor, and probing the underside of a leaf for 3 s—at which time the female is removed from the plant before laying any eggs. We define rejection as failing to perform this complete behavioral sequence during a 2-min trial. In practice, since hosts are offered at discrete points in time, we can never know exactly when a female crosses the motivational threshold that causes her to switch from rejecting to accepting any given host. Therefore, rather than reporting the exact d-phase, we report the *minimum* d-phase. This is the time from the first acceptance of the preferred host to the last rejection of the less preferred host; we know that the motivational switch for the preferred host happened at some point before the first accept and that the switch for the unpreferred host happened at some point after the last reject.

We tested *between* species host preference (*Psem* versus *Ctor*) using live, naturally growing *Psem* plants and live, potted *Ctor* plants. We tested females from 8 CC, 8 CP, 17 PC, and 11 PP families. Most butterflies were tested multiple times, and we assigned females the longest of their replicate minimum d-phases before averaging these values across sisters within families. By convention, d-phases are expressed in units of time relative to the first acceptance of *Ctor*. Therefore, d-phases for females that accepted *Ctor* before *Psem* (i.e., who preferred *Ctor*) are positive values while those for females that accepted *Psem* first are negative. The effect of cross type on these values was analyzed in a one-way ANOVA.

We tested the *within* species host preference for immature, budding *Ctor* versus mature, blooming *Ctor* using plants that we grew from seed in a greenhouse at constant temperature. The two types of *Ctor* were treated exactly the same way except that the budding plants were germinated 1–2 wk later than the blooming plants. We tested females from 3 CC, 3 CP, and 5 PC families. By convention, d-phases associated with a preference for budding plants are positive values while those associated with a preference for blooming plants are negative. Data were compiled within females and across sisters as described above for between species preference.

#### Oviposition site height

We placed oviposition motivated females, one at a time, at the top of a *Psem* plant (≥5 cm above the ground) and allowed them to freely explore the plant and choose an oviposition site. We recorded the height of the chosen sites and averaged first across replicates for individual females (anywhere between 1 and 10 replicates) and second across sisters within families. We tested females from 4 CC, 9 CP, 15 PC, and 13 PP families. We used an ANOVA to examine the effect of cross type on family averages.

#### Clutch size

We recorded the number of eggs laid by female butterflies on *Psem* and *Ctor* during multiple, staged encounters with the same, live plants used in the preference tests described above. The number of eggs laid by a female on any particular host is a function not only of the intrinsic physiological controls in which we were interested but also of the amount of time that has passed since she first became motivated to lay (the longer the time, the more eggs she has matured and the more she will likely lay). We therefore only considered clutches laid by females who had recently become motivated to lay, or more precisely who had rejected the host less than 1 h previously. If a female never rejected a given host plant despite being given frequent opportunities to lay, we considered her intrinsic clutch size for that host to be the minimum size of all clutches laid on that host within 15 min of her most recent oviposition event. We were forced to do this because females with very small intrinsic clutch sizes almost never reject their preferred host; it is difficult to catch them at a point when they don't have enough mature eggs in their abdomen to motivate them to oviposit. Females with larger intrinsic clutch sizes will wait longer periods of time after laying their previous clutch before becoming motivated to lay again (an entire day in the case of *Psem*-adapted females). We tested females from 7 CC, 9 CP, and 13 PC families on *Ctor* and 6 CP, 9 PC, and 9 PP families on *Psem*. We used an ANOVA to examine the effect of cross type on family averages for each host separately.

#### Fitness effects of clutch size and preference for budding versus blooming Ctor

We used a three-way factorial field experiment to examine the effects of maternal clutch size and host preference on the fitness of young larvae on *Ctor*. At each of 15 study sites, we identified and marked 14 young, still budding *Ctor*, and 14 more mature, blooming *Ctor*. Approximately 10–14 d later (when we would have expected eggs laid on the original plants to begin hatching), we returned and placed neonate C and P larvae (from TR and RM, respectively) on both types of plants in seven groups of size 1, 1, 5, 5, 10, 15, and 25. By this time, the original budding and blooming plants were blooming and senescing, respectively. In summary, the two types of larvae were crossed with the two types of plants and the seven group sizes for a total of 28 plants per site. We left the larvae in the field for ∼10 d before returning to count and weigh survivors. In cases where we found the plants mostly or completely defoliated (*n* = 14 of 400 plants), we guessed that absent larvae had actually left the plant in search of food rather than having died. In support of this conclusion, we sometimes found wandering larvae on non-experimental plants located near defoliated test plants. We seldom, however, found such wanderers on non-experimental plants located near test plants with edible leaf material remaining. Since the emigration of larvae from defoliated plants could bias analyses of survival, we excluded such plants from our survival analyses. We do not expect this behavior to bias analyses of weight since we could not detect a difference between the mean weight of larvae remaining on a defoliated test plants and the mean weight of wanderers collected on nearby non-experimental plants (*p* = 0.3, unpublished data). We used a multifactorial ANOVA to examine the effects of study site, larval origin (C versus P), plant age (bud versus bloom), and clutch size (1 versus 5 versus 10 versus 15 versus 25) on raw weight and arcsine-transformed survival. We excluded interaction terms from the final analyses because they were insignificant in preliminary analyses.

#### Fitness effects of clutch size on Psem

We collected naturally laid *Psem*-adapted eggs from RM, divided them into multiple groups of 5, 10, 20, or 40 eggs, and allowed each group to hatch, feed, and begin spinning a web on a small, freshly cut *Psem* leaf in a 2 mL Eppendorf tube for 24 h. We then took the tubes into the field and assigned each to a different *Psem* plant at each of 15 sites. Each site included 4–5 plants: 1–2 plants each with 5 neonate larvae, one plant with 10, one with 20, and one with 40 neonate larvae. Instead of transferring the larvae directly from the Eppendorf tubes to their assigned plants, we wedged each tube into the sandy soil beside its plant at an angle so that the opening was pointing towards the plant and almost flush with the ground. We then inserted the tip of one of the plant's basal leaves into the tube. This gave the larvae an opportunity to crawl out of the tube onto the plant at their own pace, as the cut leaf in the tube dried out (always within 12 h). We left the larvae there for 10 d before returning to count and weigh the survivors. Raw survival percentages and log-transformed weights were analyzed in separate ANOVAs that included the effects of site and clutch size (5 versus 10 versus 20 versus 40).

#### Fitness effects of oviposition site height on Psem

We caught wild P females at RM and manipulated them to lay clutches of ∼20–50 eggs on one low leaf (∼0–4 cm) and one high leaf (∼6–15 cm) from 40 naturally growing *Psem* plants in the field. In most cases, the high and low clutches for a single plant were laid by different females, but they never differed in size by more than 10 eggs. The *Psem*-adapted females readily laid on low leaves since that is their preference. To trick them into laying on high leaves as well, we pinned the leaves to the ground with a stick for the initiation of oviposition, and then allowed them to spring back up after the clutch was complete. We checked the eggs in the field every 2–3 d and recorded the date on which they hatched. We used an ANOVA to estimate the effects of plant (replicate) and level (low versus high) on development time (number of days to hatching) for all plants that had surviving eggs in both the low and high clutches (*n* = 17).

### Adaptive Surface Estimation and Phenotype Simulations

#### Estimating the shape of the fitness surface

We used the results of our field experiments to estimate the expected survival of the offspring of females with particular combinations of host species preference (*Ctor* versus *Psem*) and clutch size phenotypes. First, we fit monotone cubic splines to the survival-by-clutch size data collected on *Ctor* and *Psem* separately using the R function smooth.monotone (see grey lines in Figure 5A and 5B, right-hand panels). These give the expected survival of various clutch sizes on each host. Second, we chose a tangent function to translate the length of a female's discrimination phase (her host preference) into the probability of her choosing and laying her eggs on a particular host species. In particular, given a female's d-phase, *d* (in days),
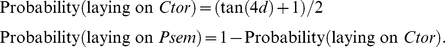
We chose this function because it has the biologically realistic property of transitioning rapidly from 0 to 1 for d-phases between −3 h and +3 h (Figure S3; see next paragraph). Finally, we calculate the expected survival of a female's offspring to be the average of the expected survival of her clutch size on *Ctor* and the expected survival of her clutch size on *Psem*, weighted by her probability of laying on each respective host given her d-phase. Rather than averaging the absolute survival probabilities on each host, we used relative survival probabilities—relative to the survival of the optimum clutch size for that host. We did this because EPI is typically thought of as a decrease in hybrid fitness *relative* to the parental types. The alternative surface based on absolute survival probabilities is shown in Figure S4.

We consider the tangent function (described above and illustrated in Figure S3) to be biologically realistic for the following reason. Females search for host plants while flying using visual cues [74]. Once they alight, they then decide whether or not to lay eggs based on chemical cues. It is the latter decision that we examine in this article and that we describe using the discrimination phase. Whether or not a female will lay on the host she prefers chemically thus depends on whether she alights on it before the end of her discrimination phase (i.e., before she becomes willing to accept the less preferred host). If she does, she will lay her eggs and reset her motivational “clock” to the beginning of her d-phase before starting her next flight search. Edith's checkerspot females alight on hosts frequently in nature (often several per minute) and usually encounter both *Ctor* and *Psem* regardless of which they prefer chemically [40],[74],[75]. In preliminary tests we found the same to be true of within- and between-host hybrid females (unpublished data). Thus, a butterfly with a 3-h d-phase is highly likely to encounter her preferred host within 3 h of initiating her search, and therefore highly unlikely to ever lay on the less preferred host.

#### Placing female butterflies on the fitness surface

We programmed a Bayesian MCMC to infer the distributions of clutch size and host preference phenotypes of female butterflies based on observed data from our oviposition behavioral assays. The ultimate goal was to simulate realistic positions for CC, CP/PC, and PP females on the adaptive surface (i.e., to simulate their oviposition phenotypes) while taking the uncertainty in our observed data into account. First, we assumed that clutch size and discrimination phase were gamma and beta distributed, respectively. A beta distribution, rather than a normal distribution, was chosen for the discrimination phase because females with d-phases>1.5 d sometimes died before finishing their tests, forcing us to truncate all observed d-phase measurements at 1.5 d before estimating the distribution. This should not affect our results since the probability of laying on the less preferred host approaches zero well before d-phases become as long as a day or two (previous paragraph, Figure S3). Second, for the observed clutch size and d-phase data from a given class of females, we programmed a Bayesian MCMC to walk through the parameter space of the geometric and beta distributions, spending the most time near parameters with high probability given the data. Prior probabilities for the distribution parameters were assumed uniform, and new parameter proposals were made by drawing random, normally distributed deviations from the current state. The variance of the normal distribution from which these random deviates were drawn was adjusted such that new proposals were accepted 20%–80% of the time. We allowed the chain to run for 1 million generations and sampled it every 1,000 generations once it had achieved stationarity. Third, for each set of gamma and beta parameters sampled from the MCMC, we randomly drew one clutch size phenotype and one d-phase phenotype and plotted their position on the adaptive surface. In essence, we simulated females of a certain type (e.g., CC, CP/PC, or PP) by drawing oviposition behaviors from the distributions that the Bayes MCMC simulation told us were mostly likely given our observed data for females of that type.

### Intrinsic Postzygotic Isolation

#### Larval growth and survival on Castilleja

To examine the fitness of pure and hybrid larvae in a “neutral” host environment that minimizes the ecological problems hybrids may face due to intermediate phenotypes that are neither fully *Psem*-adapted nor fully *Ctor*-adapted, we reared insects on *Castilleja applegatei*. We chose *Castilleja* because separate analyses of mtDNA and nuclear DNA suggest that it is the ancestral host genus of *E. editha* [39],[68]. We reared 10 neonate larvae from multiple C, CC, CP, PC, PP, and P families on cut *C. applegatei* stems standing upright in Eppendorf tubes of water for 10 d and used an ANOVA to examine the effect of cross type on log/arcsine transformed mean weight/survival at the end of the experiment.

#### Other factors

We used a non-parametric Kruskal-Wallis test to compare the viabilities of CC, CP, PC, and PP eggs laid in captivity by the mothers mated as described under “Matings.” We also examined variation in mean female teneral weight (a surrogate for fecundity) and sex ratio among hybrid families that produced adults in the summer of 2006 using an ANOVA and Kruskal-Wallis test, respectively. To assess the fertility of various types of individuals, we recorded the fertilization/viability rates of the eggs that they parented. All *E. editha* eggs are bright yellow or yellow-green when first laid. Unfertilized or inviable eggs remain this color and never hatch. Fertilized and viable eggs change color and become pink, purple, or brown within 48 h of development. We recorded the color of eggs in the first clutch parented by hybrid and pure mothers and fathers as either remaining yellow (i.e., sterile/inviable) or changed. Our practice of mating same-host hybrid females to different-host hybrid males and vice versa prevented us from comparing color change in eggs laid by the two types of hybrid parents directly. Instead, we used the color change phenotype to compare the fertility of the hybrid parents to that of pure, wild-caught individuals held in captivity over the same period of time.

## Supporting Information

Figure S1 **Early larval performance on** ***Ctor*** **(A) and** ***Psem*** **(B).** Pure and same-host hybrid insects grew and survived at indistinguishable rates (compare C versus CC and P versus PP). Different-host hybrids grew slightly more slowly on *Psem*, although the effect was only significant when predators were excluded. Colored bars show LS means ± SEM. Numbers inside bars indicate number of independent sibling groups tested. Capital letters above bars show Tukey's HSD levels for comparisons that were significant.
(0.23 MB PDF)

Figure S2 **No sign of intrinsic incompatibilities in hybrids.Different-host hybrids showed no evidence of reduced fitness in terms of egg viability, early larval growth rate on a “neutral” host, adult female teneral weight, sex ratio, or fertility.** Plots show means ± SEM for various cross types. Both same- and different-host hybrid families had mean egg viabilities of over 90% with no difference among cross types (Kruskal-Wallis *p* = 0.2; data not shown). (A) Growth rate of neonate larvae over a 10-d period on *Castilleja applegatei*. Overall variation was significant when all six cross types were compared (ANOVA *p* = 0.01), but no pairwise contrasts were individually significant by Tukey's HSD. The weak trend was for one of the reciprocal different-host hybrid types to grow faster than the others, rather than slower. (B) Teneral weight (weight at eclosion) of females raised in captivity. Variation was significant (ANOVA *p* = 0.0009). The trend is not indicative of different-host hybrid-specific incompatibilities. (C) Sex ratio of individuals reaching adulthood. Variation was marginally significant (Kruskal-Wallis *p* = 0.067). The trend was inconsistent with the presence of different-host hybrid-specific incompatibilities. (D) Fertilization rates of eggs in the first clutch parented by various types of insects. The first two categories represent reciprocal crosses between same-host hybrids and different-host hybrids. Mothers kept on ice for more than 2 wk before mating were excluded. The last category represents natural field matings between individuals from the same population. Crosses between these two hybrid classes were just as fertile as those between pure, wild-caught individuals (Kruskal-Wallis *p* = 0.9). Our mating scheme prevented us from comparing the fertility of different-host hybrids to that of same-host hybrids directly.
(0.20 MB PDF)

Figure S3 **The modeled relationship between the discrimination phase (d-phase) and the probability of laying on a particular host.** Positive and negative d-phases indicate a preference for *Ctor* and *Psem*, respectively. For a given d-phase, *d* (in days), the probability of laying on *Ctor* was modeled as (tan(4*d*)+1) / 2. It is biologically realistic that females with d-phases longer than 3 h (|d|>3 h) are highly unlikely to lay on the less preferred host.
(0.73 MB PDF)

Figure S4 **Adaptive surface shown in terms of absolute survival probability.** This surface is the same as that shown in Figure 6 except that its height reflects expected offspring survival in absolute terms rather than expected offspring survival relative to that of the optimal clutch size on each respective host (see Methods).
(1.80 MB PDF)

Table S1 **ANOVA tables from analyses of early larval performance on** ***Ctor*** **.**
(0.08 MB PDF)

Table S2 **ANOVA tables from analyses of early larval performance on** ***Psem*** **.**
(0.08 MB PDF)

Table S3 **ANOVA tables from analyses of larval performance on mature** ***Ctor*** **.**
(0.08 MB PDF)

Table S4 **ANOVA tables from analyses of the effects of foraging height on mature** ***Ctor*** **.**
(0.09 MB PDF)

Table S5 **ANOVA tables from analyses of the effects of foraging height on** ***Psem*** **.**
(0.09 MB PDF)

Table S6 **ANOVA tables from analyses of the effects of oviposition behavior on offspring growth (A) and survival (B) on** ***Ctor*** **.**
(0.08 MB PDF)

Table S7 **ANOVA tables from analyses of the effects of female clutch size on offspring growth (A) and survival (B) on** ***Psem*** **.**
(0.07 MB PDF)

Table S8 **ANOVA tables from analyses of the effects of oviposition site height on offspring development time on** ***Psem*** **.**
(0.08 MB PDF)

Table S9 **Summary of six studies of EPI in insects that specialize on distinct host plants.**
(0.07 MB PDF)

Text S1 **Analyses of genetic variation among** ***E. editha*** **populations that use** ***Ctor*** **and** ***Psem*** **at >400 polymorphic AFLPs.**
(0.18 MB PDF)

Video S1 **Oviposition site choice in a PP female butterfly.** Females whose parents were both adapted to *Psem* are very deliberate in choosing oviposition sites near the ground. In this typical example, a PP female is placed at the top of *Psem* and immediately dips her antennae towards the leaf, signaling her interest. Rather than laying right away, however, she then drops to a lower leaf and eventually to the ground. After failing to find host material on the ground she flies back to the top of the host and repeats the routine. On her third attempt, she finally locates the base of the plant and begins laying her eggs in an empty seed capsule 1 cm above the ground. This type of exploratory behavior results in the low oviposition site heights characteristic of *Psem*-adapted butterflies.
(7.88 MB MOV)

Video S2 **Oviposition site choice in a PC female butterfly.** Females with one parent adapted to *Psem* and the other adapted to *Ctor* do not usually take the time to explore their host and instead lay their eggs at the point where they first contact an acceptable host plant. In this example, a PC female is placed at the top of *Psem*, immediately becomes interested, curls her abdomen, and begins to lay her eggs. The lack of deliberate oviposition site choice results in the moderate to high oviposition site heights characteristic of most different-host hybrids and all *Ctor*-adapted butterflies.
(2.42 MB MOV)

## Abbreviations

*Ctor*: *Collinsia torreyi*
EPI: extrinsic postzygotic isolation
*Psem*: *Pedicularis semibarbata*
SEM: standard error of the mean
