# Supplementary figures and images for: Field Studies Reveal Strong Postmating Isolation between Ecologically Divergent Butterfly Populations
Source: PLoS Biol. 2010 Oct 26;8(10):e1000529. doi: 10.1371/journal.pbio.1000529 (PMC2964332; doi:10.1371/journal.pbio.1000529)

**A**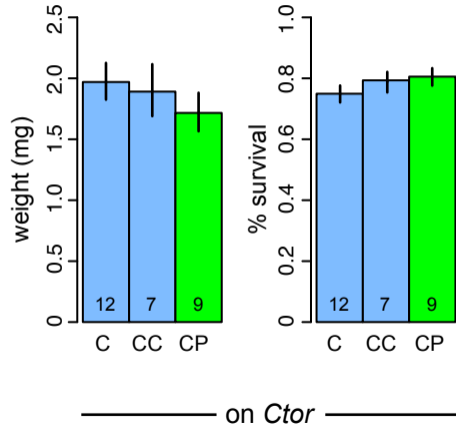**B**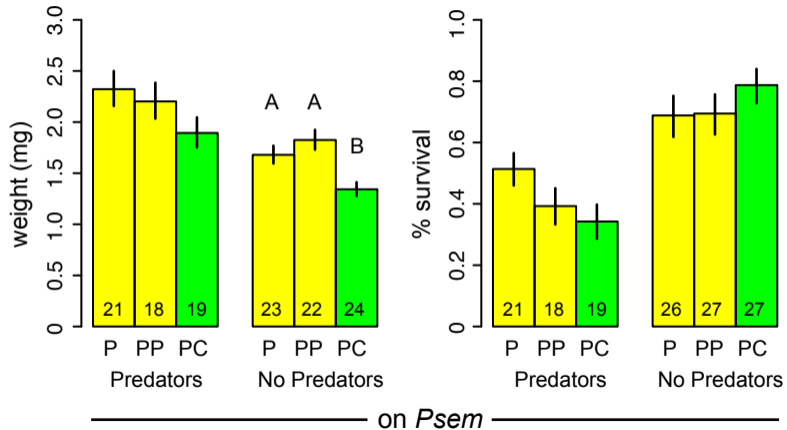

Supplement: Figure S1 — Early larval performance on Ctor (A) and Psem (B). Pure and same-host hybrid insects grew and survived at indistinguishable rates (compare C versus CC and P versus PP). Different-host hybrids grew slightly more slowly on Psem, although the effect was only significant when predators were excluded. Colored bars show LS means ± SEM. Numbers inside bars indicate number of independent sibling groups tested. Capital letters above bars show Tukey's HSD levels for comparisons that were significant. (0.23 MB PDF) [file pbio.1000529.s001.pdf]

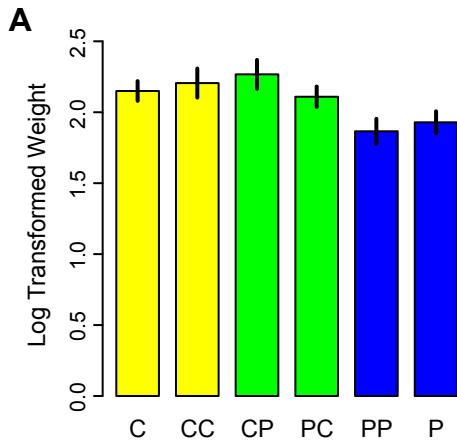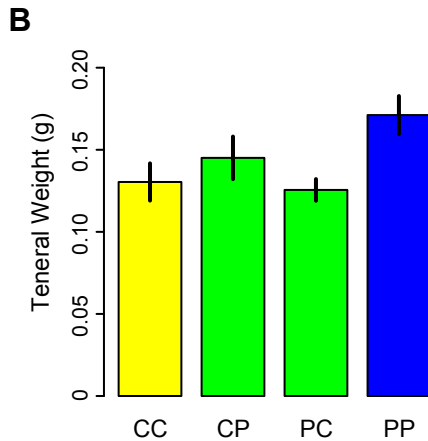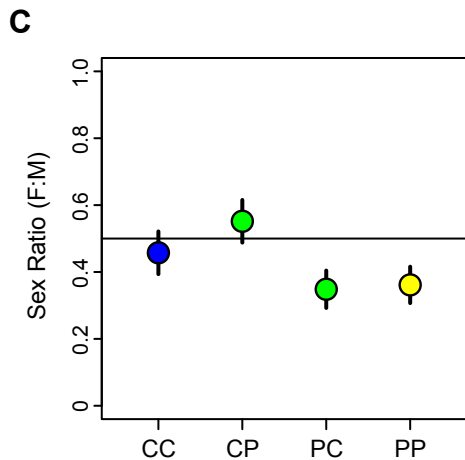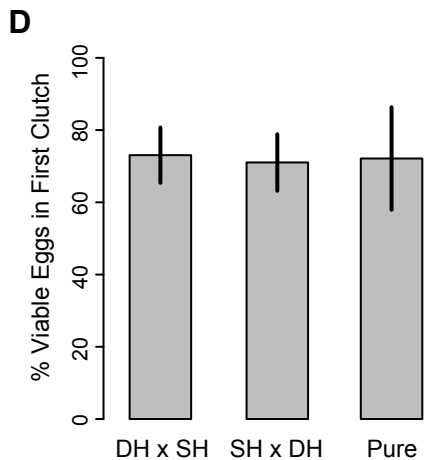

Supplement: Figure S2 — No sign of intrinsic incompatibilities in hybrids.Different-host hybrids showed no evidence of reduced fitness in terms of egg viability, early larval growth rate on a “neutral” host, adult female teneral weight, sex ratio, or fertility. Plots show means ± SEM for various cross types. Both same- and different-host hybrid families had mean egg viabilities of over 90% with no difference among cross types (Kruskal-Wallis p = 0.2; data not shown). (A) Growth rate of neonate larvae over a 10-d period on Castilleja applegatei. Overall variation was significant when all six cross types were compared (ANOVA p = 0.01), but no pairwise contrasts were individually significant by Tukey's HSD. The weak trend was for one of the reciprocal different-host hybrid types to grow faster than the others, rather than slower. (B) Teneral weight (weight at eclosion) of females raised in captivity. Variation was significant (ANOVA p = 0.0009). The trend is not indicative of different-host hybrid-specific incompatibilities. (C) Sex ratio of individuals reaching adulthood. Variation was marginally significant (Kruskal-Wallis p = 0.067). The trend was inconsistent with the presence of different-host hybrid-specific incompatibilities. (D) Fertilization rates of eggs in the first clutch parented by various types of insects. The first two categories represent reciprocal crosses between same-host hybrids and different-host hybrids. Mothers kept on ice for more than 2 wk before mating were excluded. The last category represents natural field matings between individuals from the same population. Crosses between these two hybrid classes were just as fertile as those between pure, wild-caught individuals (Kruskal-Wallis p = 0.9). Our mating scheme prevented us from comparing the fertility of different-host hybrids to that of same-host hybrids directly. (0.20 MB PDF) [file pbio.1000529.s002.pdf]

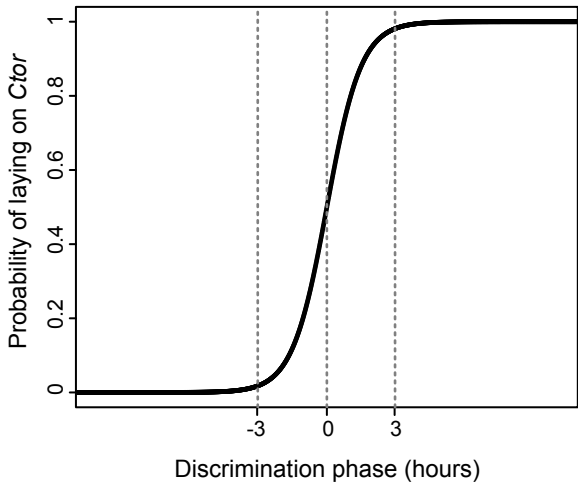

Supplement: Figure S3 — The modeled relationship between the discrimination phase (d-phase) and the probability of laying on a particular host. Positive and negative d-phases indicate a preference for Ctor and Psem, respectively. For a given d-phase, d (in days), the probability of laying on Ctor was modeled as (tan(4d)+1) / 2. It is biologically realistic that females with d-phases longer than 3 h (|d|>3 h) are highly unlikely to lay on the less preferred host. (0.73 MB PDF) [file pbio.1000529.s003.pdf]

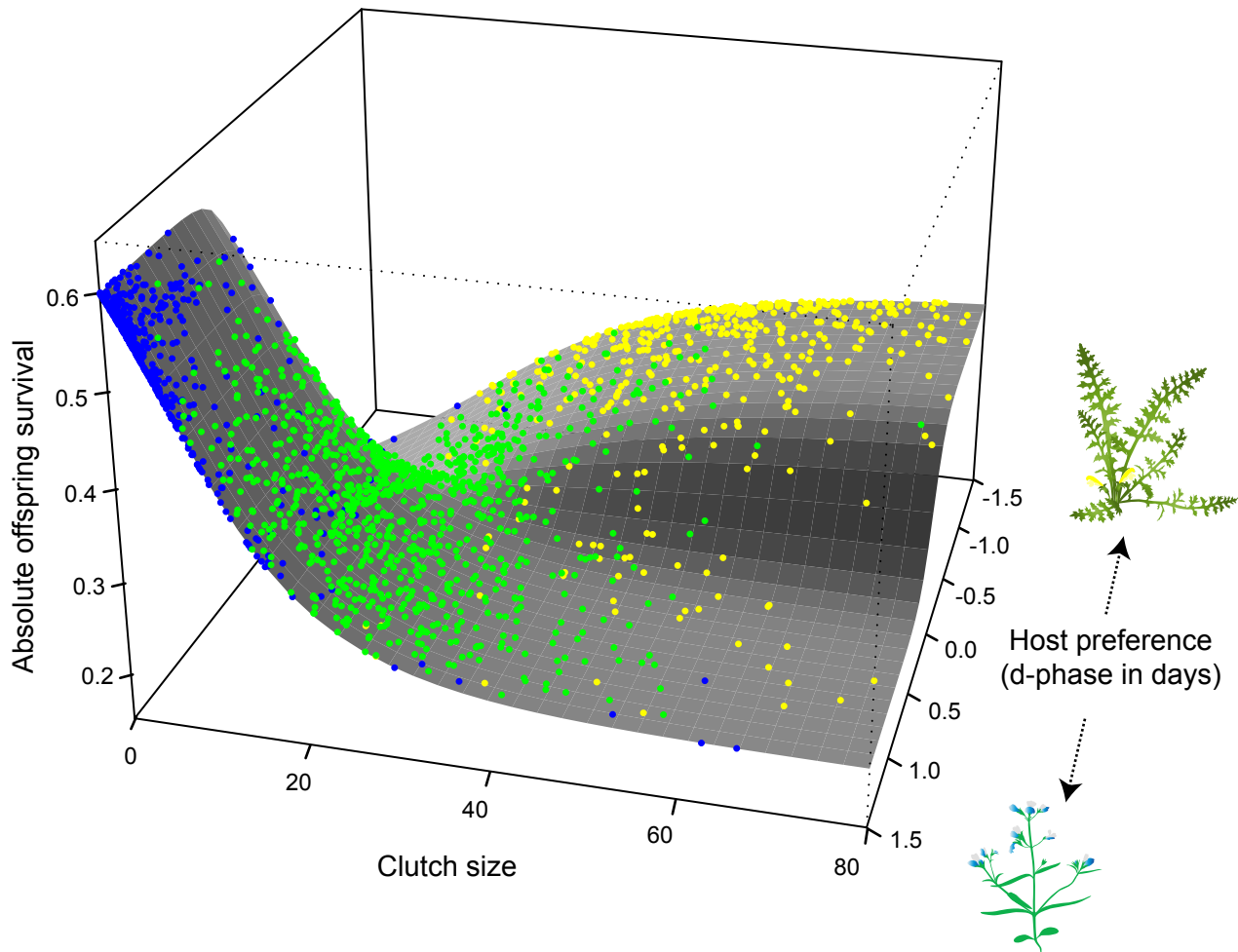

Supplement: Figure S4 — Adaptive surface shown in terms of absolute survival probability. This surface is the same as that shown in Figure 6 except that its height reflects expected offspring survival in absolute terms rather than expected offspring survival relative to that of the optimal clutch size on each respective host (see Methods). (1.80 MB PDF) [file pbio.1000529.s004.pdf]
