# Supplementary material for: Field Studies Reveal Strong Postmating Isolation between Ecologically Divergent Butterfly Populations
Source: PLoS Biol. 2010 Oct 26;8(10):e1000529. doi: 10.1371/journal.pbio.1000529 (PMC2964332; doi:10.1371/journal.pbio.1000529)
Supplement: Table S1 — ANOVA tables from analyses of early larval performance on Ctor . (0.08 MB PDF) [file pbio.1000529.s005.pdf]

Table S1. ANOVA tables from analyses of early larval performance on *Ctor*. We monitored the growth and survival of sibling groups of 4-6 individuals that were left to hatch and feed for 10 days on naturally growing *Ctor* plants in the field. The Cross Type effect encompasses overall variation among C, CC, and CP families. See Figure S1A for visual presentation of data.

A) Effects on log transformed weight

Effect	df	SS	MS	F	*P*
Cross Type	2	0.0707	0.0353	0.4512	0.6
Error	25	1.9588	0.0784		

B) Effects on arcsin transformed survival

Effect	df	SS	MS	F	*P*
Cross Type	2	0.4062	0.2031	1.5589	0.2
Error	25	3.2566	0.1303		
