# Supplementary material for: Field Studies Reveal Strong Postmating Isolation between Ecologically Divergent Butterfly Populations
Source: PLoS Biol. 2010 Oct 26;8(10):e1000529. doi: 10.1371/journal.pbio.1000529 (PMC2964332; doi:10.1371/journal.pbio.1000529)
Supplement: Table S2 — ANOVA tables from analyses of early larval performance on Psem . (0.08 MB PDF) [file pbio.1000529.s006.pdf]

Table S2. ANOVA tables from analyses of early larval performance on *Psem*. We monitored the growth and survival of sibling groups of 25-30 individuals that were left to hatch and feed for 10 days on naturally growing *Psem* plants in the field. In the experiment that included predators (A and B), each group of siblings had to be placed on a different plant to avoid mixing. Error variance therefore includes variation in plant quality (due to chemistry and/or microclimate). In the experiment that excluded predators (C and D), we were able to place one group of siblings from each of the three cross types all on a single plant. The Plant effect is therefore included in the model. The Cross Type effect examines overall variation among P, PP, and PC families. *P*-values for a priori contrasts between PC and PP families are found in the main text. See Figure S1B for visual presentation of data.

A) Effects on log transformed weight in the presence of predators

Effect	df	SS	MS	F	*P*
Cross Type	2	0.4386	0.2193	1.9159	0.16
Error	55	6.2949	0.1145		

B) Effects on arcsin transformed survival in the presence of predators

Effect	df	SS	MS	F	*P*
Cross Type	2	0.4908	0.2454	2.5798	0.08
Error	72	6.8486	0.0951		

C) Effects on log transformed weight in the absence of predators

Effect	df	SS	MS	F	*P*
Site	13	5.8438	0.4495	8.0536	< 0.0001
Plant (Site)	14	2.5841	0.1846	3.3069	0.0016
Cross Type	2	1.0567	0.5284	9.4655	0.0004
Error	39	2.1768	0.0558		

D) Effects on arcsin transformed survival in the absence of predators

Effect	df	SS	MS	F	*P*
Site	13	4.2012	0.3232	1.8402	0.06
Plant (Site)	14	3.2799	0.2343	1.334	0.2
Cross Type	2	0.3201	0.1601	0.9113	0.4
Error	50	8.7811	0.1756		
