# Supplementary material for: Field Studies Reveal Strong Postmating Isolation between Ecologically Divergent Butterfly Populations
Source: PLoS Biol. 2010 Oct 26;8(10):e1000529. doi: 10.1371/journal.pbio.1000529 (PMC2964332; doi:10.1371/journal.pbio.1000529)
Supplement: Table S3 — ANOVA tables from analyses of larval performance on mature Ctor . (0.08 MB PDF) [file pbio.1000529.s007.pdf]

Table S3. ANOVA tables from analyses of larval performance on mature *Ctor*. We monitored hybrid larvae placed on blooming to post-blooming *Ctor* plants for 10 days. We analyzed the effect of the larval type (CC *vs*. CP *vs*. PC *vs*. PP) on log transformed weight (A) and arcsin transformed survival (B). See Figure 3C for visual presentation of data.

A) Effects on log transformed weight

Effect	df	SS	MS	F	*P*
Larval Type	3	7.7878	2.5959	17.4406	<0.0001
Error	75	11.1633	0.1488		

B) Effects on arcsin transformed survival

Effect	df	SS	MS	F	*P*
Larval Type	3	0.1927	0.0642	0.4192	0.7397
Error	78	11.9508	0.1532		
