# Supplementary material for: Field Studies Reveal Strong Postmating Isolation between Ecologically Divergent Butterfly Populations
Source: PLoS Biol. 2010 Oct 26;8(10):e1000529. doi: 10.1371/journal.pbio.1000529 (PMC2964332; doi:10.1371/journal.pbio.1000529)
Supplement: Table S4 — ANOVA tables from analyses of the effects of foraging height on mature Ctor . (0.09 MB PDF) [file pbio.1000529.s008.pdf]

Table S4. ANOVA tables from analyses of the effects of foraging height on mature *Ctor*. We monitored the growth and survival of pure C and P larvae reared on the base, middle, or top sections of mature *Ctor* plants in small cups for 10 days. For each type of larvae, we analyzed the effects of plant (individual plants from which different sections were cut) and level (base vs. middle *vs*. top for P larvae; middle *vs.* top for C larvae). Analyses of growth and survival are presented in A/B and C/D, respectively. See Figure 3D for visual presentation of growth data.

A) Effects on log transformed weight of P larvae

Effect	df	SS	MS	F	*P*
Plant	14	2.3754	0.1697	3.7495	0.0022
Level	2	0.9862	0.4931	10.8965	0.0004
Error	24	1.086	0.0453		

B) Effects on log transformed weight of C larvae

Effect	df	SS	MS	F	*P*
Plant	14	2.9841	0.2132	2.7108	0.04
Level	1	1.1806	1.1806	15.0148	0.0017
Error	14	1.1008	0.0786		

C) Effects on arcsin transformed survival of P larvae

Effect	df	SS	MS	F	*P*
Plant	14	3.7097	0.2649	1.2387	0.3
Level	2	0.6238	0.3119	1.4581	0.3
Error	24	5.1339	0.2139		

D) Effects on arcsin transformed survival of C larvae

Effect	df	SS	MS	F	*P*
Plant	14	2.2966	0.164	0.9519	0.5
Level	1	0.1758	0.1758	1.0203	0.3
Error	14	2.4127	0.1723		
