# Supplementary material for: Field Studies Reveal Strong Postmating Isolation between Ecologically Divergent Butterfly Populations
Source: PLoS Biol. 2010 Oct 26;8(10):e1000529. doi: 10.1371/journal.pbio.1000529 (PMC2964332; doi:10.1371/journal.pbio.1000529)
Supplement: Table S5 — ANOVA tables from analyses of the effects of foraging height on Psem . (0.09 MB PDF) [file pbio.1000529.s009.pdf]

Table S5. ANOVA tables from analyses of the effects of foraging height on *Psem*. We monitored pure P larvae placed on high and low leaves of naturally growing *Psem* plants in the field. We analyzed the effects of plant (replicate) and height (high vs. low) on weight (A) and survival (B). For the weight analysis, we only included plants with surviving (weighable) larvae on both leaves. See Figure 3E for visual presentation of weight data.

A) Effects on log transformed weight

Effect	df	SS	MS	F	*P*
Plant	8	1.6157	0.202	3.0835	0.0659
Height	1	2.6923	2.6923	41.1049	0.0002
Error	8	0.524	0.0655		

B) Effects on arcsin transformed survival

Effect	df	SS	MS	F	*P*
Plant	15	5.9124	0.3942	5.7941	0.0008
Height	1	0.0445	0.0445	0.6543	0.4312
Error	15	1.0204	0.068		
