# Supplementary material for: Field Studies Reveal Strong Postmating Isolation between Ecologically Divergent Butterfly Populations
Source: PLoS Biol. 2010 Oct 26;8(10):e1000529. doi: 10.1371/journal.pbio.1000529 (PMC2964332; doi:10.1371/journal.pbio.1000529)
Supplement: Table S6 — ANOVA tables from analyses of the effects of oviposition behavior on offspring growth (A) and survival (B) on Ctor . (0.08 MB PDF) [file pbio.1000529.s010.pdf]

Table S6. ANOVA tables from analyses of the effects of oviposition behavior on offspring growth (A) and survival (B) on *Ctor*. We monitored the growth and survival of groups of 1, 5, 10, 15, and 25 neonate larvae placed on blooming and senescing *Ctor* plants in the field for 10 days. The Larval Type effect is included to control for differences between C larvae from Tamarack and P larvae from Rabbit Meadow, which were both used in the experiment. The Plant Phenology effect contrasts blooming and senescing plants – which would have been budding and blooming, respectively, when eggs were laid. The Clutch Size effect examines variation among the different sized groups. See Figure 5A for visual presentation of data.

A) Effects on untransformed weight

Effect	df	SS	MS	F	*P*
Block	14	18279.13	1305.65	29.613	< 0.0001
Larval Type	1	5495.21	5495.21	124.636	< 0.0001
Plant Phenology	1	1346.65	1346.65	30.543	< 0.0001
Clutch Size	4	828.59	207.15	4.698	0.0011
Error	237	10449.36			

B) Effects on arcsin transformed survival

Effect	df	SS	MS	F	*P*
Block	14	8.3076	0.5934	2.502	0.0020
Larval Type	1	0.3216	0.3216	1.356	0.2449
Plant Phenology	1	34.057	34.057	143.616	< 0.0001
Clutch Size	4	24.1869	6.0467	25.499	< 0.0001
Error	378	89.63902			
