# Supplementary material for: Field Studies Reveal Strong Postmating Isolation between Ecologically Divergent Butterfly Populations
Source: PLoS Biol. 2010 Oct 26;8(10):e1000529. doi: 10.1371/journal.pbio.1000529 (PMC2964332; doi:10.1371/journal.pbio.1000529)
Supplement: Table S7 — ANOVA tables from analyses of the effects of female clutch size on offspring growth (A) and survival (B) on Psem . (0.07 MB PDF) [file pbio.1000529.s011.pdf]

Table S7. ANOVA tables from analyses of the effects of female clutch size on offspring growth (A) and survival (B) on *Psem*. We monitored the growth and survival of groups of 5, 10, 20, and 40 neonate larvae placed on *Psem* plants in the field for 10 days. The Clutch Size effect examines variation among the different sized groups. See Figure 5B for visual presentation of data.

A) Effects on log transformed weight

Effect	df	SS	MS	F	*P*
Site	14	8.0051	0.5718	4.3388	0.0026
Clutch Size	3	0.4514	0.1505	1.1418	0.3606
Error	17	2.2404	0.1318		

B) Effects on untransformed survival

Effect	df	SS	MS	F	*P*
Site	14	2.1167	0.1512	4.2165	< 0.0001
Clutch Size	3	0.4227	0.1409	3.9295	0.0133
Error	52	1.8646	0.0359		
