# Supplementary material for: Field Studies Reveal Strong Postmating Isolation between Ecologically Divergent Butterfly Populations
Source: PLoS Biol. 2010 Oct 26;8(10):e1000529. doi: 10.1371/journal.pbio.1000529 (PMC2964332; doi:10.1371/journal.pbio.1000529)
Supplement: Table S8 — ANOVA tables from analyses of the effects of oviposition site height on offspring development time on Psem . (0.08 MB PDF) [file pbio.1000529.s012.pdf]

Table S8. ANOVA tables from analyses of the effects of oviposition site height on offspring development time on *Psem*. We monitored pure P eggs laid on high and low leaves of naturally growing *Psem* plants in the field. We analyzed the effects of plant (replicate) and height (high vs. low) on development time (time to hatching). See Figure 5C for visual presentation of development time data.

Effect	df	SS	MS	F	*P*
Plant	16	115.5294	7.2206	5.9157	0.0005
Height	1	192.9706	192.9706	158.0964	< 0.0001
Error	33	328.0294	1.2206		
