# Supplementary material for: Field Studies Reveal Strong Postmating Isolation between Ecologically Divergent Butterfly Populations
Source: PLoS Biol. 2010 Oct 26;8(10):e1000529. doi: 10.1371/journal.pbio.1000529 (PMC2964332; doi:10.1371/journal.pbio.1000529)
Supplement: Table S9 — Summary of six studies of EPI in insects that specialize on distinct host plants. (0.07 MB PDF) [file pbio.1000529.s013.pdf]

Table S9. Summary of six studies of EPI in insects that specialize on distinct host plants. Each study detected a significant reduction in F1 fitness, assayed in terms of larval growth/ survival (five studies) or lifetime fecundity (one study). Deficits were extracted from figures or tables in the primary references and averaged across host plants.

Insect taxon	Larval Growth Deficit	Larval Survival Deficit	Lifetime Fecundity Deficit	Study Setting	Ref.
Eurosta solidaginis (goldenrod gall-fly)	-	54%	-	field cages	1
Acyrthosiphon pisum (pea aphid)	-	-	47%	laboratory	2
Galerucella nymphaeae (water lily leaf beetle)	-	20%	-	laboratory	3
Mitoura nelsoni & muiri (hairstreak butterflies)	8%	16%	-	laboratory	4
Acrocercops transecta (leaf-mining moths)	-	50%	-	laboratory	5
Neochlamissus bebbianae (leaf beetles)	20%	-	-	laboratory	6

1. Craig TP, Horner JD, Horner JK (1997) Hybridization studies on the host races of *Eurosta solidaginis*: Implications for sympatric speciation. Evolution 51: 1552-1560.

2. Via S (1999) Reproductive isolation between sympatric races of pea aphids. I. Gene flow restriction and habitat choice. Evolution 53: 1446-1457.

3. Pappers SM, Van der Velde G, Ouborg NJ, Van Groenendael JM (2002) Genetically based polymorphisms in morphology and life history associated with putative host races of the water lily leaf beetle, *Galerucella nymphaeae*. Evolution 56: 1610-1621.

4. Forister ML (2005) Independent inheritance of preference and performance in hybrids between host races of *Mitoura* butterflies (Lepidoptera : Lycaenidae). Evolution 59: 1149-1155.

5. Ohshima I (2008) Host race formation in the leaf-mining moth *Acrocercops transecta* (Lepidoptera : Gracillariidae). Biol J Linn Soc 93: 135-145.

6. Egan SP, Funk DJ (2009) Ecologically dependent postmating isolation between sympatric host forms of *Neochlamisus bebbianae* leaf beetles. Proc Natl Acad Sci U S A 106: 19426-19431.
