# Supplementary material for: Field Studies Reveal Strong Postmating Isolation between Ecologically Divergent Butterfly Populations
Source: PLoS Biol. 2010 Oct 26;8(10):e1000529. doi: 10.1371/journal.pbio.1000529 (PMC2964332; doi:10.1371/journal.pbio.1000529)

Text S1. Analyses of genetic variation among *E. editha* populations that use *Ctor* and *Psem* at >400 polymorphic AFLPs.

Wee [1] analyzed genetic variation among 30 *E. editha* populations. In this supporting section, we summarize a subset of Wee's analyses focusing on 7 populations that feed on either *Ctor* or *Psem* (*Ctor* populations = LK, TR; *Psem* populations = MT, RM, MK, BM, PI; see Figure 1B of main text for geographic locations). These summaries are given in parts (A) and (B) on the next two pages. DNA was extracted from adult legs/wings or larval heads using a Qiagen DNeasy Tissue Kit. AFLP fragments were generated using a modified Applied Biosystems (ABI) protocol and analyzed on an ABI 3100 Genetic Analyzer. The three ABI primer pairs used were EcoRI-ACA Fam/MseI-CAT, AcoRI-ACA Fam/MseI-CTG, and EcoRI-AAG Joe/MseI-CTG. 546 bands were polymorphic among all 30 populations examined. >400 bands were polymorphic among individuals from the 7 *Ctor* or *Psem* feeding populations.

1. Wee B (2004) Effects of geographic distance, landscape features and host association on genetic differentiation of checkerspot butterflies. Ph.D. thesis University of Texas at Austin.

2. Schneider S, Roessli D, Excoffier L (2000) *ARLEQUIN 2.0. A Software for Population Genetic Data Analysis*. Genetics and Biometry Laboratory, Department of Anthropology, University of Geneva, Switzerland.

3. Hintz (2001) NCSS and PASS. Number Cruncher Statistical Systems. Kaysville, Utah. http://ncss.com.

4. Nei M, Li WH (1979) Mathematical model for studying genetic variation in terms of restriction endonucleases. Proc Natl Acad Sci U S A 76: 5269-5273.

(A) Populations that use the same host are no more closely related than populations that use different hosts based on Φ_{ST} estimates. Populations are listed in the table in order from northernmost to southernmost (see Figure 1B of main text for exact geographic locations). Columns 2 and 3 show the host affiliation and number of individuals genotyped for each population. Columns 4-10 show pairwise Φ_{ST} values estimated using the AMOVA module in the program ARLEQUIN 2.0 [2], which partitions genotypic variance rather than allelic variance when used with dominant markers such as AFLPs. Estimates for pairs of populations that use different hosts are highlighted in red font. The mean Φ_{ST} for different-host pairs (0.139) was no greater than that for same-host pairs (0.131; *t*-test P = 0.7).

Population	Host	n	MT	LK	TR	RM	MK	BM	PI
MT	*Psem*	3	-----						
LK	*Ctor*	11	0.11	-----					
TR	*Ctor*	17	0.13	0.08	-----				
RM	*Psem*	22	0.12	0.17	0.11	-----			
MK	*Psem*	12	0.14	0.19	0.12	0.04	-----		
BM	*Psem*	14	0.21	0.18	0.11	0.08	0.08	-----	
PI	*Psem*	9	0.16	0.15	0.12	0.17	0.19	0.17	-----

(B) Populations that use *Ctor* and *Psem* are intermingled on a non-metric multidimensional scaling (NMDS) plot summarizing the first two major axes of genotypic variation. Blue and yellow circles represent populations adapted to *Ctor* and *Psem*, respectively. Grey circles represent populations that use other host plants. The plot was produced using the program NCSS [3] based on a matrix of Nei's corrected average pairwise differences [4] generated from AFLP genotypes using the program ARLEQUIN 2.0 [2]. The first and second axes account for 26% and 13% of overall genotypic variation, respectively.

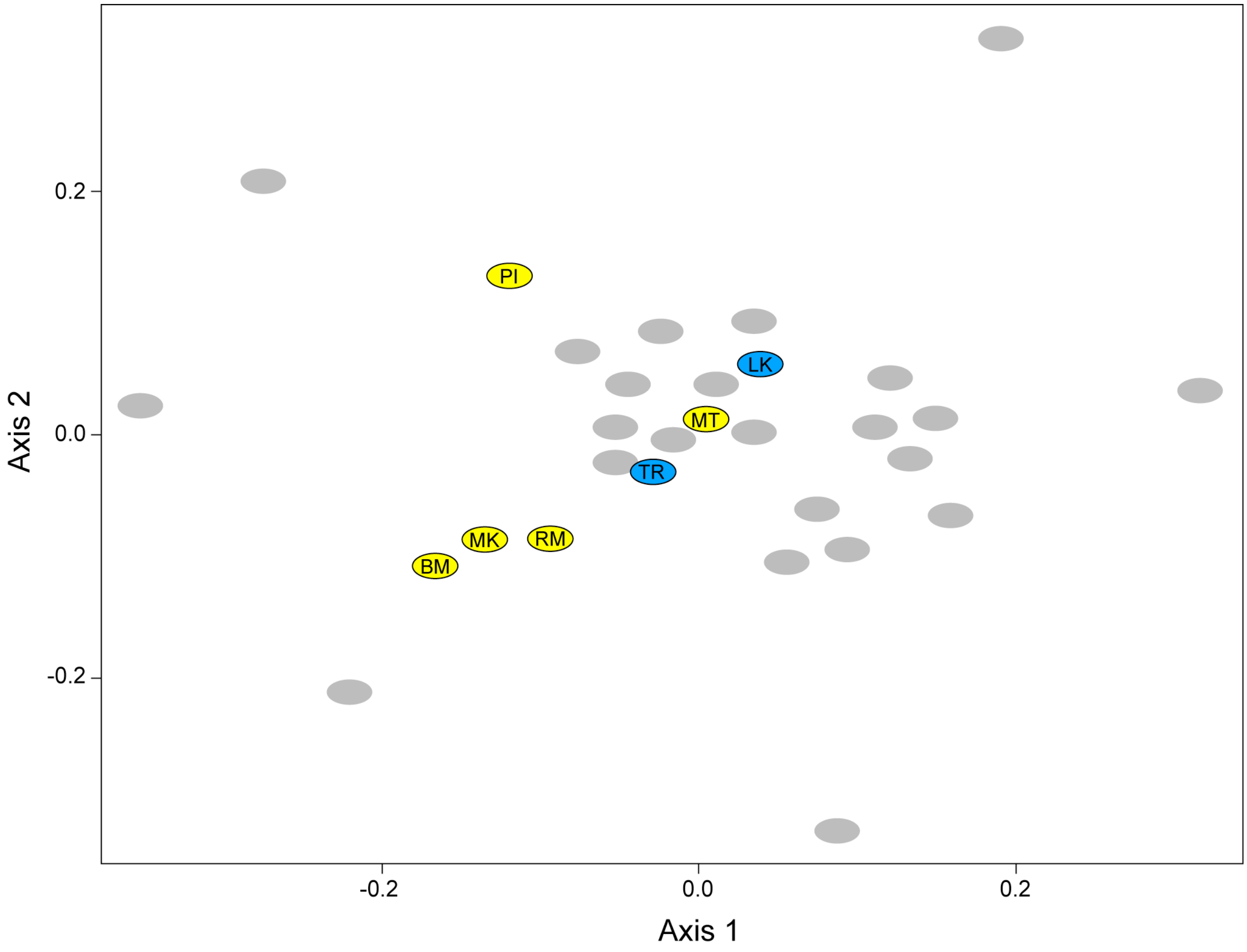

Supplement: Text S1 — Analyses of genetic variation among E. editha populations that use Ctor and Psem at >400 polymorphic AFLPs. (0.18 MB PDF) [file pbio.1000529.s014.pdf]
